# Pituitary Gonadotropin Gene Expression During Induced Onset of Postsmolt Maturation in Male Atlantic Salmon: *In Vivo* and Tissue Culture Studies

**DOI:** 10.3389/fendo.2022.826920

**Published:** 2022-03-15

**Authors:** Diego Crespo, Kai Ove Skaftnesmo, Erik Kjærner-Semb, Ozlem Yilmaz, Birgitta Norberg, Sara Olausson, Petra Vogelsang, Jan Bogerd, Lene Kleppe, Rolf B. Edvardsen, Eva Andersson, Anna Wargelius, Tom J. Hansen, Per Gunnar Fjelldal, Rüdiger W. Schulz

**Affiliations:** ^1^ Research Group Reproduction and Developmental Biology, Institute of Marine Research, Bergen, Norway; ^2^ Research Group Reproduction and Developmental Biology, Institute of Marine Research, Austevoll Research Station, Storebø, Norway; ^3^ Reproductive Biology Group, Division Developmental Biology, Department Biology, Science Faculty, Utrecht University, Utrecht, Netherlands; ^4^ Research Group Reproduction and Developmental Biology, Institute of Marine Research, Matre Research Station, Matredal, Norway

**Keywords:** puberty, pituitary, Atlantic salmon, follicle-stimulating hormone, transcriptomics

## Abstract

Precocious male maturation causes reduced welfare and increased production costs in Atlantic salmon (*Salmo salar*) aquaculture. The pituitary produces and releases follicle-stimulating hormone (Fsh), the gonadotropin triggering puberty in male salmonids. However, little is known about how Fsh production is regulated in Atlantic salmon. We examined, *in vivo* and *ex vivo*, transcriptional changes of gonadotropin-related genes accompanying the initial steps of testis maturation, in pituitaries of males exposed to photoperiod and temperature conditions promoting maturation (constant light and 16°C). Pituitary *fshb*, *lhb* and *gnrhr2bba* transcripts increased *in vivo* in maturing males (gonado-somatic index > 0.1%). RNA sequencing (RNAseq) analysis using pituitaries from genetically similar males carrying the same genetic predisposition to mature, but differing by responding or not responding to stimulatory environmental conditions, revealed 144 differentially expressed genes, ~2/3rds being up-regulated in responders, including *fshb* and other pituitary hormones, steroid-related and other puberty-associated transcripts. Functional enrichment analyses confirmed gene involvement in hormone/steroid production and gonad development. In *ex vivo* studies, whole pituitaries were exposed to a selection of hormones and growth factors. Gonadotropin-releasing hormone (Gnrh), 17β-estradiol (E_2_) and 11-ketotestosterone (11-KT) up-regulated *gnrhr2bba* and *lhb*, while *fshb* was up-regulated by Gnrh but down-regulated by 11-KT in pituitaries from immature males. Also pituitaries from maturing males responded to Gnrh and sex steroids by increased *gnrhr2bba* and *lhb* transcript levels, but *fshb* expression remained unchanged. Growth factors (inhibin A, activin A and insulin-like growth factor 1) did not change *gnrhr2bba*, *lhb* or *fshb* transcript levels in pituitaries either from immature or maturing males. Additional pituitary *ex vivo* studies on candidates identified by RNAseq showed that these transcripts were preferentially regulated by Gnrh and sex steroids, but not by growth factors, and that Gnrh/sex steroids were less effective when incubating pituitaries from maturing males. Our results suggest that a yet to be characterized mechanism up-regulating *fshb* expression in the salmon pituitary is activated in response to stimulatory environmental conditions prior to morphological signs of testis maturation, and that the transcriptional program associated with this mechanism becomes unresponsive or less responsive to most stimulators *ex vivo* once males had entered pubertal developmental *in vivo*.

## Introduction

The pituitary gland is a key organ integrating both intrinsic and extrinsic stimuli to regulate vertebrate physiology, including for example reproduction, growth or stress (1, 2). The anterior pituitary (*pars distalis* of the adenohypophysis) contains various cell types, together producing a range of peptide hormones. Among those cell types, gonadotropes produce the key regulators of reproduction, the gonadotropins follicle-stimulating hormone (FSH) and luteinizing hormone (LH) (3), which are produced by two distinct gonadotrope cell types in teleost fish (4). Gonadotropins are released into the bloodstream and regulate gonadal hormone and germ cell production (4). Extensive research carried out in numerous fish species has shown the complexity (5) but also conserved aspects of some of the main signaling systems regulating expression, synthesis and secretion of pituitary gonadotropins in teleosts, such as hypothalamic gonadotropin releasing-hormone (Gnrh) (6–9), sex steroids (7, 10, 11) and the activin/inhibin system (7, 12). Notably, androgenic and estrogenic sex steroids exert complex feedback effects on the teleost pituitary, depending among others on gender, stage of maturation, or species examined [as reviewed by Fontaine et al. (13)]. In both Atlantic salmon (*Salmo salar*) (14) and coho salmon (*Oncorhynchus kisutch*) (11), *in vivo* treatment with testosterone (T) −and with 17β-estradiol (E_2_) in the latter species− stimulated the pituitary expression of *lhb* but not of *fshb*. Similarly, in coho salmon, pituitary protein levels of Fsh remained unaffected, whereas Lh levels increased in response to T and E_2_ (11). Further studies using gonad-intact and gonadectomized Atlantic salmon parr showed that plasma Fsh levels decreased after implanting aromatizable and non-aromatizable androgens (15), demonstrating an important role of androgens in mediating negative feedback on Fsh release.

In Atlantic salmon, pituitary *fshb* expression and plasma 11-ketotestosterone (11-KT) levels both increase when males enter puberty, which is accompanied by an increase in single germ and Sertoli cell proliferation activity in the testis (14, 16). Fsh and androgen plasma levels remain elevated throughout the testicular growth period in spring/early summer (17, 18), probably reflecting the potent steroidogenic activity of Fsh (19–23). After all, Lh cannot be detected in plasma samples in salmonids until close to the spawning season in the fall, when spermatogenesis has been completed and the spermatogenic tubules are filled with mature sperm. This scenario argues for a pivotal role for Fsh regarding the regulation of entering puberty and sustaining spermatogenesis during the prolonged testicular growth period in salmonid fish. Yet, we are far from having a clear picture of the regulation of Fsh production and release in teleosts, particularly when considering the question how concomitantly increasing/elevated Fsh and androgen levels are possible in the light of a negative androgen feedback on Fsh in salmonids (10, 11).

Farmed Atlantic salmon males exposed to growth-promoting light and temperature regimes can enter precocious maturation, which negatively impacts fish welfare, growth rates and their immune status in turn causing important production losses (24). In order to gain knowledge on how to avoid entrance into precocious puberty, we investigated transcriptomic changes in the pituitary entering puberty. As a first step, we confirmed a prominent pituitary expression of *fshb* over *lhb* already prior to and even clearer after entering puberty. We also found that exposing fish to stimulatory photoperiod conditions increased the proportion of males entering puberty, which always was associated with elevated *fshb* mRNA and circulating androgen levels. Secondly, with two types of experiments we elucidated changes in the pituitary transcriptome accompanying the initiation of puberty in males, namely by (i) an RNAseq study comparing pituitaries from males about to enter puberty with immature counterparts with the aim to identify puberty-associated signals; and (ii) primary tissue culture studies using pituitaries obtained from immature or maturing males that were exposed *ex vivo* to known regulators of pituitary gene expression, such as Gnrh, sex steroids or growth factors, followed by quantifying a selection of candidate gene transcripts. The latter experiments also allowed to address the question, how a concomitant increase in Fsh and androgen plasma levels may be possible.

## Material and Methods

### Fish Maintenance and Tissue Sampling

Atlantic salmon postsmolts used in this study were reared under standard conditions and sampled at Matre Aquaculture Research Station (Matredal, Norway). All experiments herein have been approved by the Norwegian Animal Research Authority (NARA, permit number 5741); use of the experimental animals was in accordance with the Norwegian Animal Welfare Act of 19th of June 2009. Four groups of fish were studied:

*Group 1*. After smoltification, immature males (14-15 months old) were exposed to 12 hours dark/12 hours light and 16°C (referred to as non-stimulatory conditions) for a period of 16 days. Feeding was done with standard commercial diets. Prior to sampling for body weight, length and gonad weight, all fish were anesthetized with 2 mL/L Finquel vet and sacrificed by cutting into the *medulla oblongata*. The condition factor (K) was calculated as K = body weight × body length^−3^ × 100. Pituitaries from the sampled fish were cultured *ex vivo* (see *Pituitary Tissue Incubations* sections) and collected after 9 days of incubation in RNAlater (Thermo Fisher Scientific) for RNA extraction. Additional pituitaries were fixed in 4% glutaraldehyde overnight at 4°C and embedded in plastic (Technovit 7100; Kulzer) for subsequent histological analysis, as previously described (25, 26).

*Group 2*. Immature postsmolt males (14-15 months old) were exposed to continuous light and 16°C (referred to as stimulatory conditions) for 16 days, as previously described by Fjelldal et al. (27). In response to this regime, part of the fish had started pubertal development, as indicated by gonado-somatic index (GSI) levels above 0.1% and the presence of type B spermatogonia, respectively [the germ cells/cysts were identified according to previously published morphological criteria (28)]. Feeding and sampling were performed as mentioned above for *Group 1* with minor changes. Briefly, pituitaries from immature and maturing postsmolts were collected before or after *ex vivo* incubation for 9 days and stored in RNAlater until RNA extraction. Testis tissue was fixed in 4% glutaraldehyde overnight at 4°C and embedded in Technovit 7100 for histological evaluation or collected in RNAlater for RNA extraction. Moreover, plasma samples were collected for 11-KT quantification.

*Group 3*. An additional batch of (14-15 months old) immature postsmolt males was exposed to stimulatory conditions for 7 weeks. Feeding, sampling and pituitary incubations were performed as described above for *Group 2*. No testis tissue samples were collected in this experiment.

*Group 4*. All-male siblings, heterozygous for the puberty-associated *vgll3* locus (29, 30), were produced by crossing a double haploid XX female with a YY supermale (31). At the age of 14-15 months, the fish were exposed to stimulatory conditions for 6 months. At 7 and 11 days after starting the maturation regime, the fish were sampled for body weight, length, gonad weight and plasma. Pituitaries were collected and flash frozen in liquid nitrogen for high-throughput RNA sequencing analyses. A testis tissue sample was fixed in 4% glutaraldehyde, embedded in Technovit 7100 and sectioned at 3 µm thickness, and analysis of the stage of maturation showed that all males in *Group 4* exhibited testes containing type A spermatogonia as the furthest developed germ cell type, i.e. were classified as immature regarding germ cell development (28). Males were selected for sequencing analysis based on 11-KT plasma levels and on *fshb* transcript levels in the pituitary.

### Pituitary Tissue Incubation

A previously established primary tissue culture system developed for zebrafish (*Danio rerio*) (32) was used, which is also similar to the system developed for Japanese eel (*Anguilla japonica*) pituitaries (33), except that we used an agar cylinder instead of elder pith as support for pituitary tissue. For salmon pituitary incubations, the temperature was set at 14-16°C. First, in order to study the morphological and functional integrity of pituitary tissue after *ex vivo* culture, whole pituitaries collected from immature postsmolt males were incubated in L15-based medium for 3 and 9 days and subsequently fixed in 4% glutaraldehyde for histological analysis. Additional pituitaries were incubated in the absence or presence of E_2_ and collected in RNAlater for gene expression analyses.

To study the effects of potential regulators on gene expression *ex vivo*, pituitaries from immature and maturing postsmolts were incubated for 9 days in the absence or presence of different compounds (Gnrh, E_2_, 11-KT, inhibin A, activin A and Igf1). After the incubation period, pituitaries were collected for gene expression analyses. E_2_ and 11-KT were purchased from Sigma-Aldrich and used at a final concentration of 100 ng/mL (33) and 100 nM (34), respectively. Gnrh salmon analog [1 µM (8)] was purchased from Syndel. Recombinant human inhibin A and activin A proteins were purchased from Thermo Fisher Scientific and used at the same final concentration of 50 ng/mL (35, 36); the respective salmon proteins were not available commercially. Also salmon Igf1 was not available, so that we used recombinant gilthead sea bream Igf1 (37) (50 ng/mL) from ProSpec.

### Production of an Antiserum Against Atlantic Salmon Fsh

Two types of single-chain recombinant hormones were produced in CHO cells after transfection of the respective coding sequences in pcDNA3.1(+) vector (Invitrogen) by Rara-Avis (Valencia, Spain): (i) single-chain recombinant salmon Fsh (referred to as rFsh-A) consisting of Atlantic salmon Fsh beta (Fshb) subunit (acc. no. XM_014126338), a (Gly-Ser) spacer sequence and rabbit common alpha (CGA) subunit (acc. no. AF318299), and (ii) single-chain recombinant salmon Fsh (referred to as rFsh-B) consisting of Atlantic salmon Fshb subunit (acc. no. XM_014126338), a (Gly-Ser) spacer sequence and Atlantic salmon common alpha (Cga) subunit (acc. no. BT056856). The percentage identity between amino acid sequences of rabbit CGA and Atlantic salmon Cga is 70%. Yields of rFsh-A and rFsh-B, after IMAC purification, were 1.37 mg per 8x10^8^ CHO cells and 1.89 mg protein per 4x10^8^ CHO cells, respectively. Both recombinant hormones contained a TEV protease cleavage site followed by a 6X His-Tag (used for affinity purification) at the C-terminus of the protein. Antiserum was raised in rabbits against the rFsh-A protein (Agrisera AB, Vännäs, Sweden). The specificity of the Fsh antiserum was confirmed by Western blotting and immunohistochemistry (see below).

### Western Blot

Samples containing 0.25 µg of the single-chain rFsh-B were diluted in lithium dodecyl sulfate sample buffer (NuPAGE LDS Sample Buffer, Invitrogen) with 100 mM DTT (reducing conditions). Pituitaries from 3 maturing male salmon (GSI = 0.78 ± 0.05%) were mixed and homogenized in lysis buffer (10 mM Tris-HCl pH 7.5, 150 mM NaCl, 0.05% Tween 20) containing 1:100 protease inhibitor cocktail (Merck) with 104 mM AEBSF, 80 μM aprotinin, 4 mM bestatin, 1.4 mM E-64, 2 mM leupeptin and 1.5 mM pepstatin. The homogenate was incubated on ice for 10 min before being centrifuged at 14000 g for 10 min at 4°C. All samples were denatured for 10 min at 70°C and separated on Any kD Mini-PROTEAN TGX gels (Bio-Rad) at 120 V for 1 hour. A gel replicate for Coomassie blue staining was fixed in 40% ethanol/10% acetic acid for 15 min, stained overnight at room temperature with QC Colloidal Coomassie Stain (Bio-Rad) and de-stained in deionized water for 3 hours before signal detection. rFsh-B (0.25 µg) and 10 µg pituitary homogenate were separated on gels and then transferred to Trans-Blot Turbo Mini nitrocellulose membranes (Bio-Rad) at 25 V for 7 min on a Trans-Blot Turbo system (Bio-Rad). Blots containing transferred protein were blocked in 1X casein solution (Vector laboratories, Bionordika) for 10 min at room temperature and were then incubated for 1 hour at room temperature with the anti-Fsh serum, anti-Fsh serum preabsorbed with rFsh-B (at 1:28 molar ratio overnight at 4°C) or pre-immune serum, all diluted 1:5000 in PBST with 1% BSA. After washing in PBST, the membranes were incubated with biotinylated goat anti-rabbit IgG (H+L) secondary antibody (1:1000; Life Technologies) at room temperature for 2 hours and signal was developed using VECTASTAIN ABC-AmP reagent (Vector laboratories) and detected using iBright FL1500 imaging system (Invitrogen).

### Immunohistochemistry

**Figure 1:**
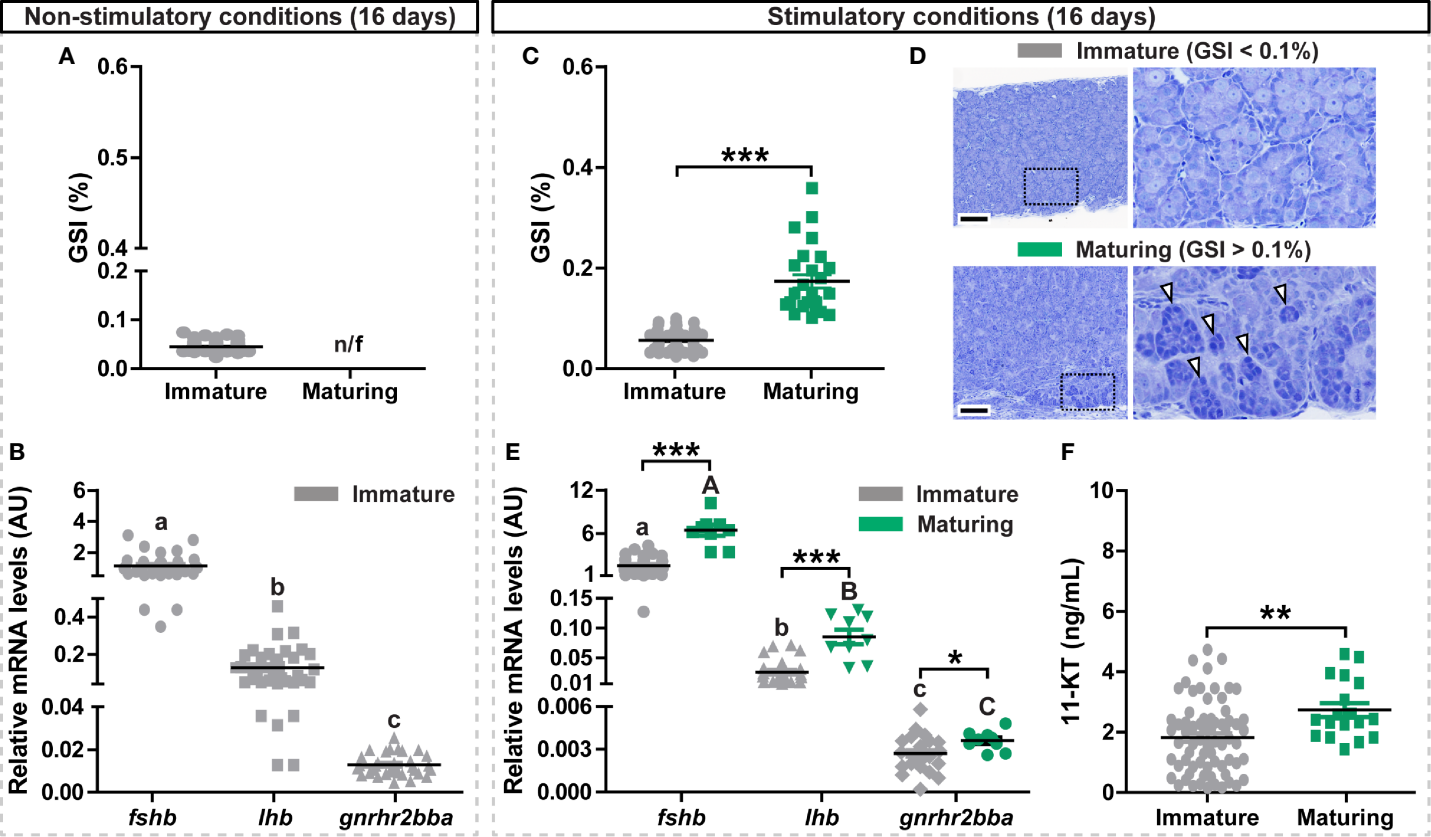
Evaluation of gonadal and pituitary responses to non-stimulatory and stimulatory conditions. **(A, B)** Gonado-somatic index (GSI; **A)** and *in vivo* expression levels of selected pituitary genes (*fshb*, *lhb*, *gnrhr2bba*; **B)** in immature postsmolt males exposed to non-stimulatory conditions (12 hours dark/12 hours light, and 16°C) for 16 days. **(C–F)** GSI **(C)**, representative testis histology **(D)**, *fshb*, *lhb*, *gnrhr2bba* expression levels **(E)** and 11-ketotestosterone (11-KT) plasma levels **(F)** in immature and maturing males after exposure to stimulatory conditions (constant light and 16°C) for 16 days. In **(A, C, F)**, data are shown as mean ± SEM (N = 18-96; **p < 0.01; ***p < 0.001) and, in **(B, E)**, shown as mean ± SEM (N = 9-35; *p < 0.05; ***p < 0.001; different letters denote significant differences between groups) and expressed relative to the *fshb* mRNA abundance. n/f, not found. AU, arbitrary units. In **(D)**, boxes identify the testis tissue areas shown at higher magnification, white arrowheads indicate representative groups of type B spermatogonia. Scale bar, 100 µm.

Pituitaries of immature and maturing male salmon (collected from a subset of fish as presented in **Figures 1C–F**) were fixed in 4% paraformaldehyde, embedded in paraffin, and serial 4 μm thick sagittal sections were prepared for fluorescent immunohistochemistry. Briefly, slides were heated using a 2100 Retriever (Electron Microscopy Sciences) for 20 min in sodium citrate buffer pH 6.0 for antigen retrieval and subsequently cooled down at room temperature for 2 hours. After washing in PBS and blocking with 5% BSA (in PBS), consecutive slides were incubated at 4°C overnight with polyclonal rabbit anti-(Atlantic salmon)Fsh (Agrisera; this study) and rabbit anti-(coho salmon)Lh antibodies (kindly provided by Dr. Penny Swanson), diluted 1:5000 in 1% BSA/PBS. Then, slides were incubated with the secondary antibody (mouse anti-rabbit IgG Alexa Fluor 488; Life Technologies) at room temperature for 90 min. Propidium iodide (Sigma-Aldrich) was used as nuclear counterstain and slides were mounted with SlowFade Diamond Antifade Mountant (Thermo Fisher Scientific). Pituitary sections were analyzed using a standard fluorescence microscope (Nikon Eclipse 80i).

Digital images from fluorescent stained sections were subjected to a quantification pipeline. First, a pixel classification of the nuclei was performed with Ilastik (38) to separate nuclei from background. Using the probability maps produced in Ilastik, nuclei were then segmented in CellProfiler (39–41) and propagation outward from the nuclei was used to locate the cell boundaries. Cell cytoplasm was located as the cell boundary minus the nuclei boundary, and any positive signal inside the cytoplasm was measured by the ‘‘MeasureObjectIntensity’’ and ‘‘MeasureGranularity’’ modules in CellProfiler. Positive cells were then scored by filtering on median intensity and granularity of the staining. Four non-overlapping fields of the *proximal pars distalis* per individual and antibody (anti-Fsh or anti-Lh) were photographed at 400X magnification in serial pituitary sections, and the percentage of Fsh- and Lh-positive cells quantified. Therefore, the total area examined for fluorescent signal was the same for both groups.

Additional pituitary sections were examined by chromogenic staining of Fsh protein using EnVision+ Dual Link System-HRP (DAKO), according to the manufacturer’s protocol. Briefly, endogenous peroxidase activity was blocked by incubation with 0.3% H_2_O_2_ in PBS for 30 min. After incubation with the anti-Fsh serum and washing in PBST, slides were incubated with the polymer-HRP secondary antibody at room temperature for 30 min, after which slides were incubated with DAB substrate kit (VECTASTAIN, Vector laboratories) for 5 min. Nuclei were counterstained with hematoxylin and slides mounted with Histokitt (Chemi-Teknik) after dehydration.

### 11-KT Quantification by ELISA

Plasma concentrations of 11-KT were analyzed by ELISA (42) on extracted plasma samples, as previously described (43). Acetylcholine esterase-labeled tracers and microplates pre-coated with monoclonal mouse anti-rabbit IgG were supplied by Cayman Chemicals. 11-KT standard was purchased from Sigma-Aldrich. Anti-11-KT was a kind gift from David E. Kime (Sheffield University, UK).

### RNA Extraction and cDNA Synthesis

Each pituitary was collected on RNAlater for preservation and stored at 4°C until use. Individual pituitaries were homogenized in 400 µL of homogenization buffer and processed according to the Maxwell HT-simplyRNA kit instructions (Promega) on a BioMek 4000 instrument (Beckton Dickinson), and RNA was DNase treated as part of the RNA extraction procedure. The quantity and purity of RNA samples were assessed by spectrophotometry on a Nanodrop ND-1000 instrument (Thermo Fisher Scientific). cDNA was prepared by reverse transcription of 200 ng RNA using the SuperScript IV VILO Master Mix with ezDNase Enzyme (Thermo Fisher Scientific) according to the manufacturer’s recommendations.

### Quantitative Real-Time PCR (qPCR)

Except for qPCR assays previously published, primers specific for the genes of interest were designed using the BatchPrimer3 online tool (https://probes.pw.usda.gov/batchprimer3/). Primers are listed with their respective sequence and citation in **Supplementary Table 1**. A qPCR reaction was prepared according to the manufacturer descriptions to contain 800 nM of each forward and reverse primer in a 6 µL reaction containing a 1x concentration of the PowerUp SYBR Green Master Mix, or TaqMan Fast Advanced Master Mix where a TaqMan probe was required (Thermo Fisher Scientific). 2 µL of a 1/20 dilution of cDNA was added to the reaction and all qPCR assays were analyzed using a QuantStudio 5 Real-Time PCR system (Thermo Fisher Scientific). The relative gene expression level was calculated using the comparative *Ct* (2^−ΔΔCt^) method (44). For each gene, all values were normalized to *ef1a* expression.

### Transcriptomic Analysis Using RNA Sequencing

We used immature sibling salmon males for the RNAseq study, which showed a low genetic variation since they were produced by crossing a double haploid XX female with an inbreed YY supermale, created by self-fertilization from a hermaphrodite (31). These immature fish, heterozygous for the puberty-associated *vgll3* locus, were exposed to constant light and 16°C. Part of the population did (responders), another part did not (non-responders), react to these stimulatory environmental conditions by showing signs for entering puberty (see *Results* section below). Pituitaries from responders and from non-responders were used to examine transcriptomic differences. Total RNA was isolated from each pituitary using the Maxwell HT-simplyRNA kit (Promega) and quantified, as described above. RNA integrity was checked with an Agilent Bio-analyzer 2100 total RNA Nano series II chip (Agilent). Only samples with an RNA integrity number > 8 were used for library preparation. 200 ng RNA of each pituitary sample were reverse transcribed and the synthesized cDNAs were then used for *fshb* screening by qPCR, as previously described. Illumina RNAseq libraries were prepared from the remaining 1-2 µg total RNA using the Illumina TruSeq Stranded mRNA Library Prep Kit (Illumina, Inc.) according to the manufacturer’s instructions. The resulting RNAseq libraries were sequenced on an Illumina HiSeq 4000 sequencer (Illumina, Inc.) as 150 bp pair-end reads. Base calling was done by the Illumina pipeline. Quality control of the obtained reads was performed using FastQC suite (v0.11.7; default parameters). RNAseq derived reads were mapped to the salmon genome (ICSASG_v2) using Bowtie2 v2.3.5.1 (45). The resulting files were filtered with SAMtools v1.10 (46), and the read counts for each transcript were extracted using SAMtools idxstats and summed for each unique GeneID. Data analysis was performed with the R/Bioconductor package DESeq (47) (p-adjusted < 0.001). Genes were discarded if all samples had normalized read counts less than 50. The raw RNAseq data of the 10 samples sequenced (5 biological replicates per condition) have been deposited in the NCBI BioProject database with accession number PRJNA778619.

Functional enrichment analysis was carried out using the WebGestalt online tool (48) (http://www.webgestalt.org/), which calculates over-representation (FDR < 0.05) of Gene Ontology (GO) categories (49). The g:Orth option of the g:Profiler tool (50) (http://biit.cs.ut.ee/gprofiler/gost) was used to retrieve mouse (*Mus musculus*) orthologs from the list of differentially expressed genes (DEGs) and exported as the input for functional enrichment analysis (Biological Process GO category). Regulated KEGG pathways were determined using the KEGG Mapper tool (51). KEGG pathways represented by at least 3 DEGs and by the ratios of regulated genes (up-/down-, and *vice versa*) higher than 2.5 were considered for the analysis.

### Statistical Analysis

GraphPad Prism 9.0.0 package (GraphPad Software, Inc.) was used for statistical analysis. Significant differences between groups were identified using Student’s t test or one-way ANOVA followed by Tukey’s test for multiple group comparisons, as appropriate (*, p < 0.05, **, p < 0.01, ***, p < 0.001; ns, no significant changes observed). For datasets with no normal distribution, the non-parametric two-sided Mann–Whitney test was applied. The correlations between GSI values and *fshb*, *lhb* and *gnrhr2bba* expression levels, and 11-KT plasma concentration, were analyzed using Spearman’s rank test (data were Log_2_-transformed to meet homogeneity of variances). Results are represented as mean ± SEM.

## Results

### Photoperiod Manipulation Triggers Sexual Maturation and Pituitary Gonadotropin Gene Expression in Atlantic Salmon Postsmolt Males

To investigate the transcriptional changes of selected gonadotropin-related genes (*fshb*, *lhb* and *gnrhr2bba*) accompanying initial steps of testis maturation, we exposed immature postsmolt males to both non-stimulatory and stimulatory conditions. While exposure to non-stimulatory conditions (12 hours dark/12 hours light and 16°C, for a period of 16 days) did not trigger testis growth (GSI < 0.1%; **Figure 1A**), an increasing proportion of the fish entered pubertal development from day 10 when kept under stimulatory conditions (24 hours light and 16°C, for a period of 16 days) (**Supplementary Figure 1E**), as indicated by GSI levels above 0.1% and the presence of type B spermatogonia (**Figures 1C, D**). Condition factor and body weight, but not body length, were also increased in maturing males exposed to stimulatory conditions (**Supplementary Figures 1C, D**). Pituitary *fshb* expression levels were at least 10-fold higher than *lhb*, and at least 100-fold higher than *gnrhr2bba* levels in immature fish of both photoperiod regimes (**Figures 1B, E**). *fshb*, *lhb* and *gnrhr2bba* transcripts all increased in the maturing fish exposed to stimulatory conditions (**Figure 1E**), and all transcripts showed significant correlations with the GSI values (**Supplementary Figures 2B, E, H**). Remarkably, despite already showing high expression levels, the amplitude of the response to the stimulatory conditions was highest for *fshb* (4.25-fold), compared to *lhb* (2.56-fold) and *gnrhr2bba* (1.32-fold). A significant increase in plasma 11-KT was also observed in maturing fish (**Figure 1F**), and its levels correlated to GSI and *fshb* expression values (**Supplementary Figure 3**).

### Production and Validation of the Specific Antiserum for Atlantic Salmon Fsh

Considering the prominent response of *fshb* transcript levels upon entering puberty, we aimed at investigating the cellular expression of Fshb protein in Atlantic salmon pituitaries. For that purpose, a chimeric single-chain Fsh peptide (rFsh-A; containing the mature Atlantic salmon Fshb subunit, a spacer sequence and rabbit CGA common alpha subunit) was produced, and an Fsh antiserum was then raised in rabbits against the rFsh-A protein.

**Figure 2:**
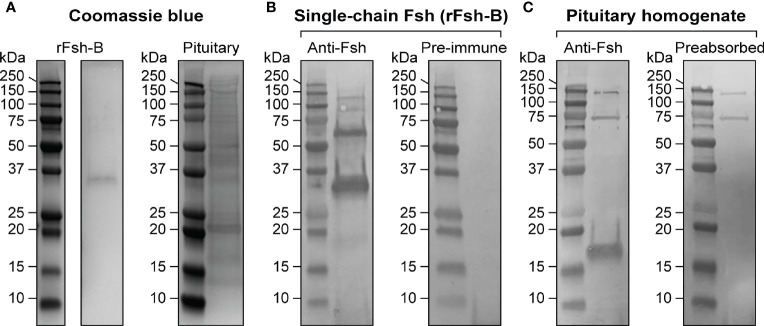
Characterization of a specific antiserum for Atlantic salmon Fsh. **(A)** Coomassie blue protein staining of the single-chain Fsh protein (rFsh-B) and of a pituitary homogenate. **(B)** Representative immunoblots of rFsh-B using Fsh antiserum (left panel) and pre-immune serum (as negative control; right panel). **(C)** Representative immunoblots of a pituitary homogenate using anti-Fsh serum (left panel) and anti-Fsh serum preabsorbed with rFsh-B (as negative control; right panel). In all blots, molecular mass markers (kDa) are shown on the left.

The Fsh antiserum was characterized by Western blotting, using pituitary extracts of maturing male salmon (GSI = 0.78 ± 0.05%) and the single-chain rFsh-B protein (Atlantic salmon Fshb subunit, a Gly-Ser spacer sequence and Atlantic salmon Cga common alpha subunit) as samples for electrophoresis. Coomassie blue protein staining (**Figure 2A**) and immunoblotting analysis (**Figure 2B**) indicated that the Fsh antiserum recognized the rFsh-B protein with molecular mass of approximately ~35 kDa. This corresponds well with the molecular weight determined by detecting His-Tags in the rFsh-B protein (**Supplementary Figure 4A**). Minor bands of approximately 18, 70, 90 and 125 kDa were also visible (left panel in **Figure 2B**). Additional bands on the Western blot could represent strongly glycosylated proteins, or degradation of the protein. None of the bands were observed using pre-immune serum (right panel in **Figure 2B**). A clear band of approximately 18 kDa was detected in a pituitary extract of maturing males, corresponding to the molecular weight of Atlantic salmon Fshb subunit (**Figure 2C**, left panel). This band disappeared when preabsorbing the Fsh antiserum with rFsh-B (**Figure 2C**, right panel). Two higher molecular weight bands (~75 kDa and ~150 kDa) were found (**Figure 2C**) when using Fsh antiserum, but also with pre-immune serum and another, unrelated antiserum raised in rabbit (data not shown), suggesting they represent non-specific staining not related to Fsh.

**Figure 3:**
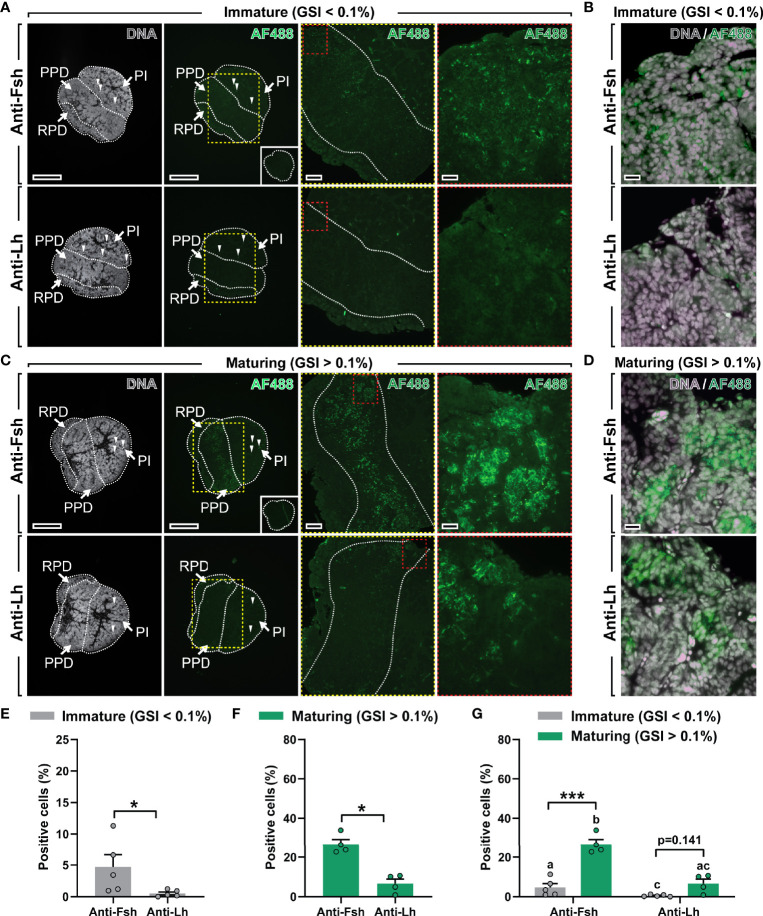
Localization and quantification of Fsh and Lh cells in immature and maturing male salmon pituitaries. **(A–D)** Immunolocalization of Fsh and Lh proteins in serial sections (sagittally oriented; anterior to the left) of immature and maturing salmon pituitaries using the Atlantic salmon Fsh antiserum generated in this study and an anti-(coho salmon)Lh antibody previously validated (18). Right panels in **(A)** and C show pituitary tissue magnified from the marked areas (yellow and red dashed lines; scale bar = 200 µm and 50 µm, respectively). Scale bar in low magnification pictures = 500 µm. Negative control (pre-immune serum) for Fsh immunostaining showed no specific staining (insets in right lower panels in **(A, C)**. Arrowheads indicate red blood autofluorescence. Further magnified areas of pituitary tissue of immature **(B)** and maturing **(D)** male salmon do not seem to show clear Fsh/Lh co-localization in serial sections (scale bar = 20 µm). Propidium iodide (in grey) was used as nuclear counterstain. GSI, gonado-somatic index; AF488, Alexa Fluor 488; PPD, *proximal pars distalis*; PI, *pars intermedia*; RPD, *rostral pars distalis*. **(E–G)** Quantification of the percentage of Fsh- and Lh-positive cells in immature **(E)** and maturing **(F)** pituitaries, and combined **(G)**. Data are shown as mean ± SEM (N = 4-5; *p < 0.05; ***p < 0.001), and expressed relative to the total number of cells. In **(G)**, different letters indicate significant differences between groups.

The Fsh antiserum was then used for immunohistochemistry on serial sections from pituitaries of immature and maturing male salmon after 16 days exposure to stimulatory conditions (collected from a subset of fish as presented in **Figures 1C–F**). In immature pituitaries, the antiserum labeled some but not many Fsh-containing pituitary cells located in the *proximal pars distalis* (PPD; **Figure 3A**). However, the number of Fsh-positive cells and the signal intensity was remarkably higher in pituitaries of maturing males, where clearly Fsh-positive cells were widely distributed in the PPD region (magnified areas from the marked yellow and red dashed lines; **Figure 3C**). No signal was observed in control sections incubated with pre-immune serum (right lower insets in **Figures 3A, C**; see also **Supplementary Figure 4B**), indicating that the non-specific staining detected by Western blotting did not result in non-specific staining patterns on tissue sections. Moreover, using an anti-(coho salmon)Lh antibody previously generated and validated (18), very few or no (in 3 out of 5 males) Lh-positive cells were identified in immature Atlantic salmon pituitaries (**Figure 3A**). Despite observing an increase in their incidence during maturation, the number (and signal intensity) of Lh cells was clearly lower compared to those observed for Fsh cells, in both immature and maturing pituitaries (**Figures 3A, C**). Quantification of the percentage of Fsh- and Lh-positive cells supported the microscopical evaluation of pituitary sections, since the incidence of Fsh cells was significantly higher, irrespective of the stage of maturation (**Figures 3E, F**). Furthermore, the percentage of Fsh cells, in contrast to Lh cells, increased during maturation (**Figure 3G**). Considering that the Fsh and Lh antisera were both raised in rabbits, we were not able to carry out co-localization studies for Fsh/Lh protein expression on a single section. While single immuno-detection of Fsh and Lh on consecutive sections did not provide evidence for co-localization, it also does not allow excluding cellular co-localization with certainty (**Figures 3B, D**).

### Environmentally Induced Changes in the Pituitary Transcriptome

**Figure 4:**
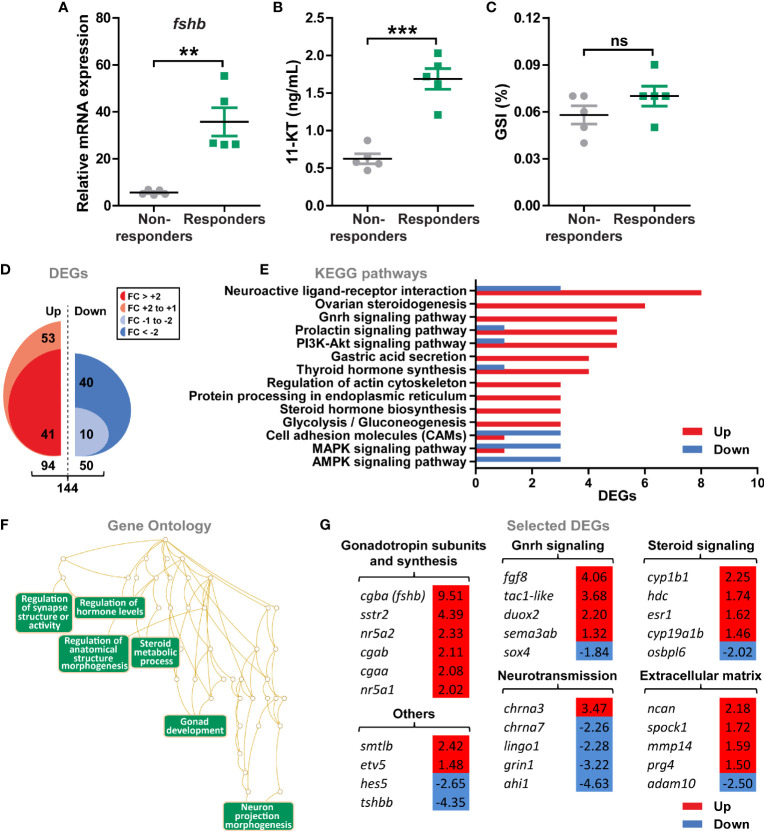
Gene expression profiling of pituitaries from male postsmolts about to enter puberty. **(A–C)** To select pituitary samples for RNAseq, sibling salmon males with low genetical variance were sampled at day 7 and 11 after exposure to stimulatory conditions (constant light and 16°C) and different puberty-associated parameters evaluated. Analysis of *fshb* expression **(A)** and 11-ketotestosterone [11-KT; **(B)**] levels revealed two groups of fish showing low (non-responders) or high (responders) levels for both parameters, while gonado-somatic indices (GSI; **C)** were unaffected. Data are shown as mean ± SEM (N = 5; **p < 0.01; ***p < 0.001). ns, no significant differences between groups. **(D)** Total numbers of up- and down-regulated genes (DEGs) identified by RNAseq (N = 5; p-adjusted < 0.001). **(E, F)** Regulated KEGG pathways **(E)** and Gene Ontology **(F)** terms in pituitaries of males responding to stimulatory conditions. KEGG pathways represented by at least 3 DEGs and ratio of regulated genes higher than 2.5 were considered for the analysis. **(G)** Selected DEGs identified by KEGG and GO analyses grouped by their function. Fold change values are shown with a red or blue background indicating up- or down-regulation, respectively.

Pituitaries from sibling salmon males with low genetic variation (31) and sharing the same genetic predisposition to mature (29, 30), were analyzed by RNAseq to investigate global gene expression of pituitary tissue upon entering puberty. To this end, juvenile males were exposed to stimulatory photoperiod and water temperature conditions (constant light and 16°C, as described above) for 11 days and samples were collected after 7 and 11 days. The pituitaries were assigned to two groups, high or low, depending on the *fshb* transcript levels (**Figure 4A**), which also correlated with high or low 11-KT plasma levels (**Figure 4B**). We refer to these two groups as responders and non-responders, respectively. It is important to note that neither GSI (**Figure 4C**) nor testicular histology (spermatogonia type A were the furthest developed germ cell type in both groups; data not shown) differed between the two groups.

144 genes were differentially expressed (DEGs) in pituitaries from responders *versus* non-responders (**Figure 4D**). Two thirds of the DEGs increased (94 or 65%), one third decreased (50 or 35%) in pituitary tissue from responding males, while the proportion of DEGs that reached a more than 2-fold change in expression was higher for the down- *versus* the up-regulated genes (80% *versus* 44%; **Figure 4D** and **Supplementary Data 1**). The majority of KEGG terms significantly enriched in pituitaries from responders contained genes that were up-regulated (**Figure 4E**), including pathways related to Gnrh, prolactin and PI3K-Akt signaling, steroid and thyroid hormone biosynthesis, as well as others involved in metabolic processes (e.g. glycolysis/gluconeogenesis) and neurotransmission (e.g. neuroactive ligand-receptor interaction). To further investigate characteristics of the pituitary transcriptome of males responding to stimulatory conditions, Gene Ontology terms were retrieved. Functionally related gene groups emerging from this analysis included factors involved in hormone (steroid) metabolism, gonad development and neuron morphogenesis (**Figure 4F**). Among the candidates identified by functional analyses, we observed that transcript levels of all gonadotropin subunits −*cgba* (*fshb*), *cgaa*, *cgab*− and genes involved in the regulation of gonadotropin synthesis (*nr5a1*, *nr5a2* and *sstr2*) were increased in pituitaries from responder males (**Figure 4G**). Also, Gnrh and steroid signaling, as well as extracellular matrix genes were preferentially up-regulated, in particular regarding estrogen signaling (*esr1*, *cyp19a1b* and *cyp1b1*; **Figure 4G**). On the contrary, a higher number of down-regulated genes was identified in the neurotransmission category (**Figure 4G**). In addition, the gene set “others” included factors previously associated to puberty and the Notch signaling pathway (*etv5* and *hes5*, respectively), as well as other pituitary hormones (*smtlb* and *tshbb*; **Figure 4G**).

### Pituitary Gene Expression Is Modulated *Ex Vivo* Before and After Entering Puberty But *fshb* Transcript Levels Are Responsive Only in Immature Males

First, we established that pituitary tissue from immature fish showed normal morphology following whole-organ tissue culture for up to 9 days (**Supplementary Figures 5A–H**). We then examined transcriptional changes of selected gonadotropin-related genes in response to E_2_ and found that *gnrhr2bba* and *lhb*, but not *fshb*, mRNA levels increased (**Supplementary Figure 5I**). These results indicate that the tissue culture system preserves structural and functional characteristics of the salmon pituitary, including gonadotroph cells.

**Figure 5:**
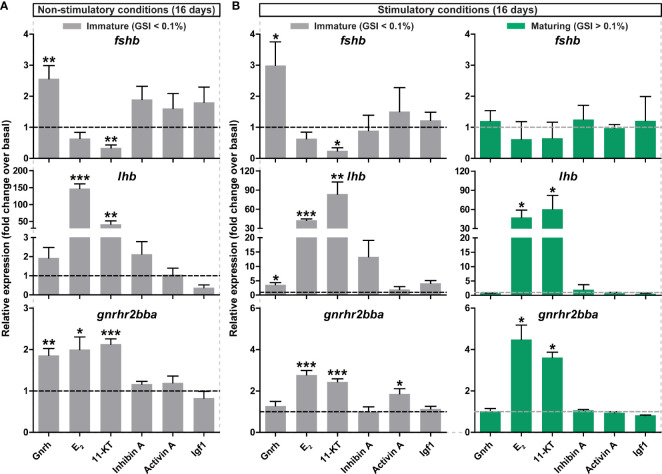
*Ex vivo* effects of potential regulators on selected pituitary genes. **(A, B)** Expression levels of *fshb*, *lhb* and *gnrhr2bba* in pituitaries collected from immature postsmolt males exposed to non-stimulatory conditions [12 hours dark/12 hours light, and 16°C; **(A)**] and immature and maturing postsmolt males exposed to stimulatory conditions [constant light and 16°C; **(B)**] for 16 days, and subsequently incubated *ex vivo* for 9 days in the presence of various potential regulators of pituitary gene expression. Results are shown as mean fold change ± SEM (N = 3-6; *p < 0.05; **p < 0.01; ***p < 0.001) and expressed relative to the control basal condition, which is set at 1 (dashed line).

By exposing immature fish to stimulatory environmental conditions, we generated sibling groups with individuals that did, or did not, enter puberty, and then used primary pituitary tissue cultures to investigate the effect of Gnrh and sex steroids −the most enriched signaling pathways identified by RNAseq during puberty− on gonadotropin gene expression in pituitaries collected from immature and maturing sibling males. We observed an up-regulation of *gnrhr2bba* and *lhb* in response to Gnrh, E_2_ and 11-KT in pituitaries of immature fish exposed to both non-stimulatory and stimulatory conditions (**Figures 5A, B**). While Gnrh also increased *fshb* transcript levels in immature pituitaries *ex vivo*, they were decreased by 11-KT (**Figures 5A, B**). As reported for immature fish exposed to non-stimulatory photoperiod conditions (**Figure 5A**), as well as for fish that remained immature despite having been kept under stimulatory conditions (**Figure 5B**; grey bars), sex steroids consistently increased *lhb* and *gnrhr2bba* transcript levels also when incubating pituitaries from fish that had started puberty (**Figure 5B**; green bars). In contrast, a Gnrh-dependent modulation in the expression of the three selected pituitary genes was not observed, and *fshb* transcript levels remained unchanged in response to all compounds tested when incubating pituitaries from maturing males (**Figure 5B**; green bars).

In addition to Gnrh and sex steroids, other potential modulators of gonadotropin gene expression such as inhibin A, activin A and Igf1 were tested *ex vivo*. However, statistical significance was only reached for *gnrhr2bba* transcript levels in response to activin A in immature pituitaries of fish exposed to stimulatory conditions (**Figure 5B**; grey bars).

### Expression Levels of Selected Candidate Genes Identified by RNAseq Are More Sensitive to Gnrh and Steroids Prior to Than After Entry Into Puberty

**Figure 6:**
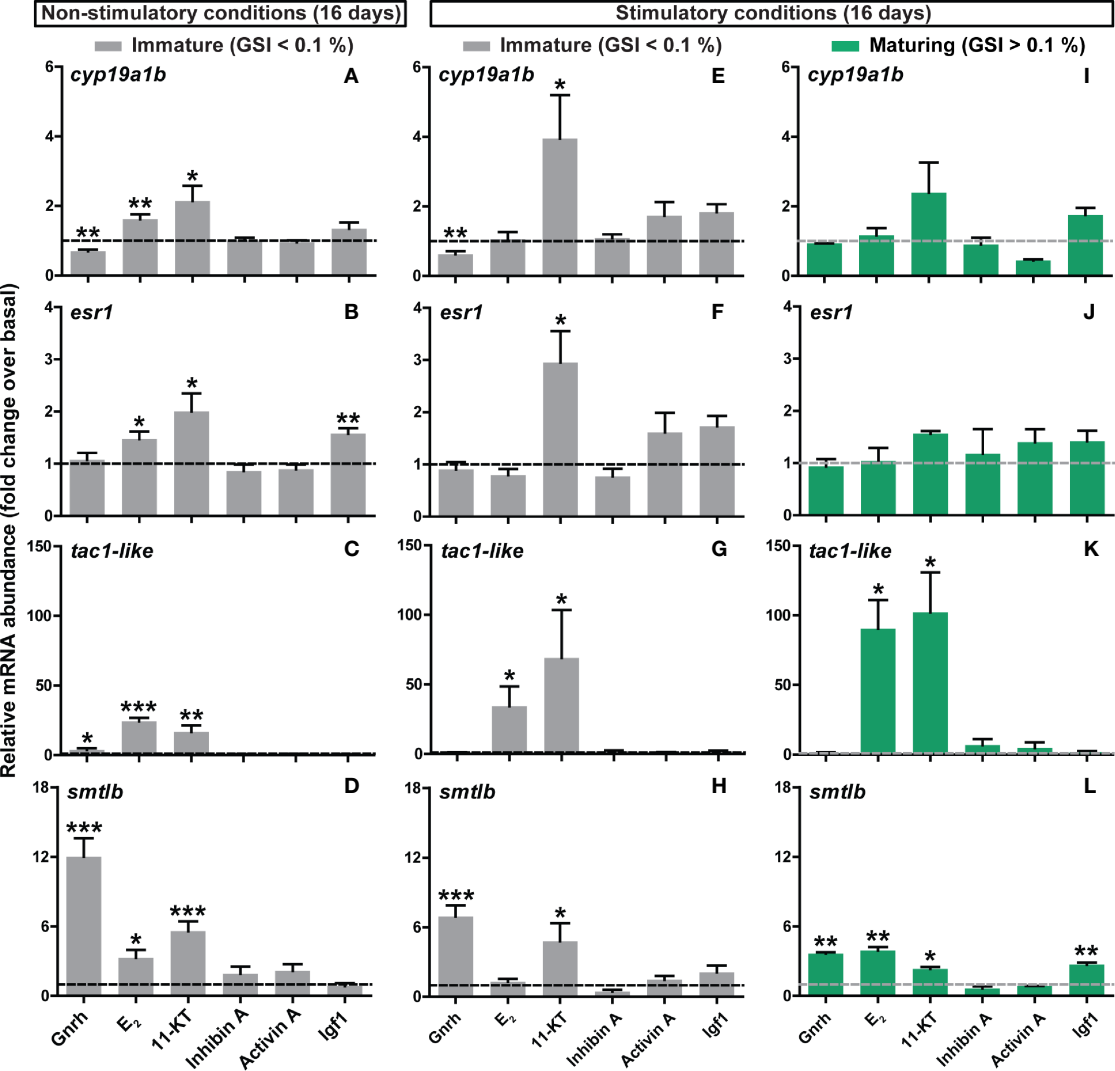
*Ex vivo* effects of different ligands on selected pituitary genes identified by RNAseq. **(A–D)** Expression levels of steroid (*cyp19a1b*, *esr1*), neurotransmission (*tac1-like*) and pituitary hormone (*smtlb*) genes in pituitaries collected from immature postsmolts exposed to standard photoperiod conditions and 16°C for 16 days, and subsequently incubated *ex vivo* for 9 days in the presence of various ligands. **(E–L)** The same set of candidate genes was analyzed in immature **(E–H)** and maturing **(I–L)** pituitary tissue after exposure to stimulatory conditions (constant light and 16°C) for 16 days, and subsequently incubated *ex vivo* for 9 days in the presence of the same ligands. Data are shown as mean fold change ± SEM (N = 4-11; *p < 0.05; **p < 0.01; ***p < 0.001) and expressed relative to the basal control group, which is set at 1 (dashed line).

We then investigated the transcriptional response of candidates identified by pituitary RNAseq upon entry into puberty as differentially expressed, examining the same array of ligands previously tested to evaluate gonadotropin gene expression *ex vivo*. Selected candidates include: (i) somatolactin (*smtlb*) and thyrotropin subunit beta (*tshbb*) pituitary hormones; (ii) genes involved in estrogen signaling (brain aromatase, *cyp19a1b*; estrogen receptor 1, *esr1*); and (iii) protachykinin-like (*tac1-like*). This selection was based on previous studies describing the importance of these factors in the regulation of sexual maturation (see *Discussion*). In immature fish kept under standard photoperiod conditions, Gnrh (in most cases) and steroids clearly modulated the expression of all candidate genes studied (**Figures 6A–D**) except of *tshbb* (data not shown). While transcript levels of *cyp19a1b*, *esr1*, *tac1-like* and *smtlb* were increased by both E_2_ and 11-KT, Gnrh up-regulated *tac1-like* and *smtlb* and down-regulated *cyp19a1b* (**Figures 6A, D**). Interestingly, *cyp19a1b* was the only candidate gene examined for which Gnrh and steroids induced an opposite effect in expression (**Figure 6A**). In addition, Igf1 increased the expression of *esr1* (**Figure 6B**).

Similar results with respect to Gnrh and steroids were found in pituitaries from males that remained immature after exposure to stimulatory conditions, however, exposure to E_2_ did not reach statistical significance, except for *tac1-like* (**Figures 6E–H**). In fish that did enter pubertal development *in vivo* in response to the stimulatory regime, only 2 of the genes investigated were modulated *ex vivo* (*tac1-like* and *smtlb*; **Figures 6I-L**). Transcript levels of *tac1-like* again showed the strong, and *smtlb* more modest, response to E_2_ and 11-KT (**Figure 6K**); steroids and Gnrh and now also Igf1 significantly up-regulated *smtlb* expression (**Figure 6L**).

These results suggest that, as observed for *fshb*, *ex vivo* changes in the pituitary transcriptome are more sensitive to regulatory signals in immature salmon males and that treatment with Gnrh and/or steroids consistently modulated pituitary gene expression.

### Long-Term Exposure to Stimulatory Conditions Results in Stronger *In Vivo* But Not *Ex Vivo* Effects

Additional functional studies were carried out to investigate changes in pituitary gene expression in response to long-term maturation trials. For that purpose, immature males were exposed to stimulatory conditions (constant light and 16°C) for 7 weeks and the incidence of maturation was scored weekly from week 4 of treatment. As expected, a higher proportion of maturing males was found compared to ~2 weeks (16 days) of exposure to stimulatory conditions (**Supplementary Figures 1E** and **6A**). Also GSI values of maturing males were clearly higher (**Figures 1C** and **Supplementary Figure 6B**) and significant increases in both body weight and length, and condition factor, were found in long-term treated fish (**Supplementary Figures 6C-E**). *In vivo* pituitary *gnrhr2bba*, more clearly *lhb*, and very clearly *fshb* transcripts all increased in maturing males kept under long-term stimulatory conditions (**Supplementary Figure 6E**). Although significant, correlations between GSI and gonadotropin gene expression were weaker in fish exposed to the long-term (**Supplementary Figures 2C, F, I**) compared to the medium-term maturation regime (**Supplementary Figures 2B, E, H**). Remarkably, pituitaries from males that remained immature despite 7 weeks of stimulatory conditions, did not respond to Gnrh or 11-KT *ex vivo* by increased *fshb* transcript levels (**Supplementary Figure 7A**), in contrast to the immature fish in the medium-term experiments (**Figure 5**). The 11-KT-mediated increase in *lhb* and *gnrhr2bba* transcript levels, on the other hand, was still observable, albeit less prominently than after ~2 weeks of stimulatory conditions (**Figures 5** and **Supplementary Figure 7**).

## Discussion

### Fsh Prior to and During Entry Into Puberty in Atlantic Salmon

The pituitary gland produces a variety of hormones regulating the development and functioning of tissues/organs involved in all major life processes in vertebrates, including the master regulators of gametogenesis and sexual maturation, the gonadotropins FSH and LH (1, 2). In fish species with long, annual reproductive cycles, analysis of plasma levels during the cycle suggest that Fsh release increases when spermatogenesis is initiated and stays elevated during the testicular growth phase while increasing/peak Lh levels appear (much) closer to the spawning season, when the spermatogenic process is already far progressed or completed. This suggests that Lh effects in males, probably mediated to a great part by very high androgen levels, are more related to spawning-related secondary sexual characters/behaviour, while Fsh drives spermatogenesis (52, 53). This scenario seems to also apply to the Atlantic salmon reproductive cycle, where *fshb* pituitary transcript levels increased during initial steps of pubertal development in males (16, 28). A homologous Fsh immunoassay is not available for Atlantic salmon to date, but Fsh plasma levels peak during sexual maturation in other salmonids (17, 18). When measuring Fsh plasma levels in Atlantic salmon using a radioimmunoassay for coho salmon Fsh, GSI and Fsh values were correlated during the main testicular growth phase in spring and summer (10). Therefore, the Atlantic salmon is an excellently suited model to specifically focus on the role of Fsh at the beginning of puberty/spermatogenesis.

Previous studies on gonadotropins in Atlantic salmon parr (54) or grilse (55), coho salmon (18) or rainbow trout (*Oncorhynchus mykiss*) (17, 56) collected samples during natural reproductive cycles. Analyzing gonadotropin blood levels, Lh is very low or undetectable while Fsh is clearly present in the circulation for the several months that most of testicular growth takes place. Furthermore, examining Fsh effects on testicular steroidogenesis (22) or gene expression (57) suggested that Fsh can induce testis maturation in salmonids. However, it is not clear what changes are taking place in the pituitary when males enter puberty. Here, we applied an experimental approach and subjected a sibling group of immature fish to environmental conditions known to promote entry into puberty, which split the treatment group into responders and non-responders. Subsequently, we focused on analyzing puberty-associated changes in pituitary gene expression.

Our main findings are: 1) when sampled one week after exposure to stimulatory conditions, changes in the pituitary were already clearly established in responders, while no morphologically discernible changes had occurred in testis tissue yet; 2) analysis of the pituitary transcriptome revealed a transcriptional network during the onset of puberty that was characterized by the regulation of different pituitary hormones and the activation of Gnrh and steroid signaling systems; 3) when sampled two or more weeks after exposure to stimulatory conditions, there was a strong correlation between the up-regulation of *fshb* in the pituitary and activation of pubertal spermatogenesis and maturing males showed increased growth; 4) the transition into puberty *in vivo* in response to stimulatory conditions blunts both, the stimulatory effect of Gnrh and the inhibitory effect of 11-KT, on *fshb* transcript levels otherwise seen as response in pituitaries collected from immature males; 5) the *fshb* transcript levels in pituitaries of males not responding to long-term exposure to stimulatory conditions *in vivo* also did not respond *ex vivo* to 11-KT or Gnrh.

Considering the pituitary hormone of central relevance for puberty, *fshb* levels largely (~10 times) exceeded those of *lhb* already in immature postsmolt males kept under standard, non-stimulatory photoperiod conditions (16, 55), and a stronger *fshb*/GSI than *lhb*/GSI correlation was found upon maturation. Here, we moreover added to the previous observations the response to stimulatory conditions (27), namely a clear up-regulation of *fshb* (4.25-fold), while *lhb* also significantly increased (2.56-fold) but showed a smaller amplitude. Both, the higher baseline expression values and the stronger up-regulatory response, support the view that Fsh is the relevant gonadotropin at the start of puberty in salmon. However, the physiological significance of gonadotropins ultimately relies on their actual plasma concentrations, so that homologous assays for plasma gonadotropins are required to further investigate their involvement in gonad development in Atlantic salmon.

In addition to studies on mRNA abundance in pituitary tissue, parallel analyses were carried out to investigate gonadotropin expression and their cellular localization at the protein level. The specific antiserum for Atlantic salmon Fsh protein generated in the current study and a previously validated anti-(coho salmon)Lh antibody (18) served to show that, akin to the respective mRNA levels, Fsh is abundant in contrast to Lh in immature salmon pituitaries. Several Fsh-positive cells were found in the PPD, while Lh-positive cells were scarce. The number of both gonadotroph cell types increased in maturing fish, though reaching statistical significance only in the case of Fsh cells in the samples analyzed here. Overall, the observed changes in protein and transcript levels agreed well with each other. The background of the strong (~5-fold) increase in the number of Fsh cells requires further studies. Previous work in juvenile African catfish (*Clarias gariepinus*) showed that an increase in Lh cell number following estrogen treatment was not associated with cell proliferation, but rather with a recruitment of pre-existing quiescent into hormone producing cells (58). In medaka (*Oryzias latipes*), on the other hand both, recruitment and proliferation was reported during ontogenesis and following estrogen exposure (59).

Previous work suggests that, different from mammals, gonadotropic hormones are produced and secreted by distinct endocrine cells in the teleost pituitary (60, 61). Studies using transgenic zebrafish and tilapia (*Oreochromis niloticus*) expressing fluorescence-tagged gonadotropes (62) and single-cell RNAseq of tilapia pituitaries (63) confirmed that Fsh and Lh are synthesized and secreted from distinct cells. However, a study in medaka that combined multi-color *in situ* hybridization (ISH) and single-cell RNAseq analyses revealed the existence of a small fraction (4%) among the gonadotrophs co-expressing *fshb* and *lhb* (64). Furthermore, in primary cell culture, medaka Fsh cells can produce Lhb (65). Considering the plasticity of gonadotropes (4), the possible existence of multi-hormonal cells cannot be ruled out for Atlantic salmon, although both transcript and protein data suggest that this is not relevant quantitatively during the initiation of pubertal spermatogenesis.

### Pituitary Gene Expression Profiling: Global Changes *In Vivo* During the Induced Onset of Puberty and *Ex Vivo* Responses of Selected Genes Towards Different Stimuli

The regulation of pituitary gonadotropin production and release is based on a network of neuroendocrine factors from the hypothalamus that also integrates signal molecules produced by peripheral organs (4, 66). However, knowledge on genes/pathways operating in the teleost pituitary during the initiation of puberty and their potential involvement in the regulation of gonadotropin production and release is still limited. A few studies have examined global changes on pituitary gene expression, comparing juvenile and adult males in model fish species (64, 67). However, no information is available on transcriptional changes in the pituitary during the narrow time window of initiating male puberty. Previous work of our group has described changes in the testis transcriptome when entering puberty in Atlantic salmon, a period characterized by increased *fshb* pituitary transcript and 11-KT plasma levels (16). Accordingly, these two parameters have been used here for assigning pituitary samples to the two groups (responders and non-responders). Our RNAseq results showed that the genes differentially expressed in the pituitary of maturing males included factors involved in Gnrh and steroid signaling, neurotransmission and extracellular matrix remodeling.

Regarding pituitary hormones, the transcript levels of *fshb*, *cgaa*, *cgab*, *smtlb* were all enhanced at the onset of maturation (*fshb* showing the highest amplitude of 9.51-fold increase), while *tshbb* expression was strongly inhibited. This pattern is in agreement with what has been described previously in zebrafish (67). Reduced *tshb* expression in maturing fish is very interesting in context with observations in zebrafish, showing that thyroid hormone potentiated Fsh-induced androgen production (68). Recent genetic evidence supported this thyroid-testis connection, considering that *tshba* mutant males neither showed secondary sex characters nor reproductive behaviour when tested with wild-type females (69). Assuming the existence of a similar thyroid-testis connection in salmon, it seems possible for example that androgens exert a negative feedback on pituitary thyrotropes. In contrast to *tshbb*, *smtlb* expression levels were enhanced *in vivo* in maturing fish in the present study. Two different forms of somatolactin (alpha and beta) were previously cloned in Atlantic salmon, and both forms increased in the pituitary during vitellogenesis and spawning in females (70). In coho salmon males, plasma somatolactin increased during the rapid testicular growth phase, was highly correlated to the 11-KT plasma concentration, and peaked during final maturation and spawning (71). Here, we found a consistent 11-KT stimulation of *smtlb* expression in pituitary cultures, irrespective of the maturational status and of the environmental conditions. While this positive feedback of 11-KT on somatolactin production in Atlantic salmon fits well to the observed changes in coho salmon androgen and somatolactin plasma levels, little is known so far regarding potential reproductive functions of somatolactin.

In both zebrafish (67) and medaka (64), but not in salmon (this RNAseq study analyzing samples collected within 1.5 weeks after starting the exposure to stimulatory conditions), *lhb* expression levels were also increased after the start of puberty. This discrepancy probably reflects the fact that those studies compared juvenile −immature− with adult −mature− stages while here, we compare two early stages of development, representing fish before and about to enter puberty, but prior to histologically visible signs of testis maturation. However, as mentioned above, *lhb* transcript abundance is elevated also in salmon when exposure to stimulatory conditions lasted longer and developmental changes have become obvious also in testis tissue, viz. the presence of type B spermatogonia and increased GSI values. This suggests that initiating puberty primarily involved activating *fshb*/Fsh expression and release, while effects on *lhb* gene expression seem secondary in nature (see below section on sex steroid effects). Next to effects on *lhb* transcript levels based on Fsh-triggered sex steroid production (see below), recent work in tilapia revealed that Gnrh-stimulated Fsh-producing cells can promote hormone synthesis in Lh-producing cells in a paracrine manner (72). However, as mentioned earlier, Lh plasma levels do not increase until much later in the salmonid reproductive cycle.

In addition to pituitary hormones, the expression levels of relevant signaling molecules involved in the regulation of gonadotropin expression and/or gonadotrope function in mammals, *nr5a1* (73, 74), *nr5a2* (75, 76) and *sstr2* (77), were also significantly elevated in the pituitary gland of maturing salmon. As one of the neuropeptides regulating gonadotropin expression and synthesis in vertebrates (78), enrichment of pituitary-expressed genes related to Gnrh signaling was expected. Among those, *tac1-like*, *fgf8*, *duox2*, *sema3ab* and *sox4* are considered as regulators of *GnRH* gene expression and/or GnRH secretion in mammals (79–83). Indeed, gene expression profiling of hypothalamic-pituitary-gonadal axis tissues identified *Sema3a* and *Fgf8* as puberty-associated factors in mice (84), and loss-of-function mutations in both genes are associated to pubertal disorders in humans (80, 85). However, in mammals, these factors mainly function in the hypothalamus while we found differential expression in the pituitary. Interestingly, in tilapia, neurokinin B (Tac3) and its receptor (Tac3r) are both expressed by Lh- and Fsh-producing cells (86), and Tac3 analogues increased pituitary *fshb* mRNA and brain *gnrh* mRNA levels (72). In mammals, the *TAC3*/*TAC3R* genes are critical for the functioning of the Kiss1-neurons in the hypothalamic arcuate nucleus, responsible for both, the negative steroid feedback as well as the pulsatile release of Gnrh (87). However, also *TAC1*/*Tac1*, encoding substance P and neurokinin A, have been implicated in the regulation of reproductive functions (88–91). *Tac1* mutant male and female mice showed delayed puberty onset (79, 92) and, conversely, exposure to TAC1R agonists triggered sexual maturation of pre-pubertal females (92). Recently, *tac1* transcript expression has been reported in the zebrafish brain but, unlike mammals, Tac1 action on Gnrh3 neurons seems independent of kisspeptin (93). In the present study, transcripts encoding different Tac and Tacr variants were detected in pituitary tissue but were not differentially expressed (data not shown), except for *tac1-like*. Future work will have to show if signaling *via* peptides derived from the *tac1-like* transcript in the pituitary is relevant for regulating gonadotroph functions in salmon. For the present study, we included the *tac1-like* paralog for further qPCR studies, since it was the only differentially expressed one among the 4 paralogs (see below).

A total of six Gnrh receptor paralogs have been identified in the Atlantic salmon genome, *gnrhr2bba* being the only paralog stimulated during precocious male parr maturation (94). The same study reported that *gnrhr2bba* transcript was solely expressed in *lhb*-expressing cells, however, a correlation between *fshb* and *gnrhr2bba* transcript levels (both increased) in earlier stages of maturation was also observed (94). Results obtained in other fish species support Gnrhr expression by Fsh-cells: (i) a recent single-cell RNAseq study in tilapia found cell-specific enrichment of *gnrhr1* in Fsh cells; (ii) in zebrafish, a close association (direct contact) was observed between Fsh cells and Gnrh3 axons (95). Until confirmation by other techniques than ISH, and studies at other life stages than parr, one should not yet dismiss the possibility of direct Gnrh action on Fsh-cells in salmon. In any case, the published work in different fish species suggest several options for stimulating Fsh-cells, such as direct effects, indirect effects *via* Gnrhr on Lh-cells, but also *via* other (non Gnrh) peptides, such as the Tac family (96). Irrespective of the mechanism(s) used by Gnrh, earlier studies in pre-pubertal male coho salmon showed that Gnrh increased *fshb* transcript levels as well as Fsh release from primary pituitary cell cultures (6). We confirmed the stimulatory effect on *fshb* transcript levels in the present study when culturing pituitary tissue from immature Atlantic salmon males, and moreover found that Gnrh treatment also increased *gnrhr2bba* transcript levels. Thus, in immature males, Gnrh seems to prime Fsh-cells to respond to subsequent Gnrh stimuli, thereby triggering in an auto-stimulatory manner increased Fsh production at the onset of puberty. Since positive feedback systems require inhibitory loops to avoid overstimulation, it was interesting to note that when exposure to stimulatory conditions had activated spermatogenesis to proceed to producing type B spermatogonia, the *fshb* response to Gnrh in culture was blunted. This can be understood by assuming that Gnrh-mediated signaling was already fully stimulated in the more progressed fish, so that their pituitaries were unable to respond to additional stimuli in culture. Alternatively, the more progressed testicular state of development may have blunted the Gnrh effect specifically regarding Fsh. We favour the latter hypothesis, since Gnrh kept stimulating somatolactin (*smtlb*) transcript levels in pituitary tissue from fish showing type B spermatogonia. Finally, Gnrh only increased *gnrhr2bba* transcript in immature fish exposed to non-stimulatory conditions, and exposure to stimulatory conditions that did not result yet in producing type B spermatogonia already blunted the Gnrh-induced *gnrhr2bba* response, which potentially contributes to preventing overstimulation of Fsh-cells. Taken together, it appears that Gnrh signaling plays a self-stimulatory role during the initiation of puberty to kick-start Fsh-production, but that this role may be limited in time, in view of the blunting of the effect on *gnrhr2bba* or *fshb* transcript levels once males have experienced stimulatory environmental conditions or commenced the production of type B spermatogonia, respectively. Hence, we assume that the exposure to stimulatory environmental conditions triggers central-nervous processes upstream of Gnrh, resulting in increased hypophysiotropic Gnrh release.

Gonadal sex steroids are key molecules controling gonadotropin production and release in vertebrates. Due to the limited availability of Fsh assays for fish species, information on the regulation of Fsh release is relatively scarce, compared to Lh. Sex steroid effects on *lhb* gene expression can be mediated by estrogen receptors and their use of estrogen response elements in the *lhb* promoter/enhancer region (34, 97, 98), but probably also by androgen receptors (15, 99, 100). Despite the essential role of androgens, such as T and 11-KT in male reproductive physiology also in fish (101–103), not a single gene annotated as being androgen-specific, was significantly regulated in our RNAseq study. Literature describing cell type-specific androgen receptor expression at the pituitary level is not available in salmonids to date, but in tilapia three different androgen receptor paralogs were enriched specifically in Fsh cells (63). Evidence for a direct effect of androgens comes from studies, in which non-aromatizable, 11-oxygenated androgens increased *lhb* transcript levels in three teleost species [Atlantic salmon parr (15), African catfish (99) or stickleback (100)]. Our present experiments confirmed this stimulatory effect of 11-KT on *lhb* transcript levels and moreover revealed, that this effect is (i) a direct one on the pituitary level, and (ii) an invariable response not depending on the maturational state or the environmental conditions. Regarding the initiation of puberty, on the other hand, this response of *lhb* transcript levels seems to be of little acute physiological relevance, considering the previously discussed, non-detectable or very low Lh plasma levels in salmonid fish during the testicular growth phase. However, 11-KT is known to exert negative feedback effects on Fsh release in coho (11, 104) and Atlantic salmon (15). We also found a negative feedback effect of 11-KT quantifying *fshb* transcript levels in our studies, and moreover saw that this inhibitory effect was characteristic of immature males but disappeared after the start of spermatogenesis, i.e. this inhibitory effect is restricted to immature fish. Importantly, this may explain the apparently paradoxical observation of concomitantly increasing Fsh and 11-KT plasma levels, despite the negative feedback effect of 11-KT on Fsh release previously reported by others in salmonids (see above). Moreover, once Fsh release has induced the start of spermatogenesis *via* modulating Sertoli growth factor (105) and Leydig cell androgen production, including the main teleost androgen 11-KT (22), the elevated 11-KT levels invariably increase pituitary *gnrhr2bba* transcript levels. We assume that the consistent up-regulation of Gnrh receptor expression in combination with the disappearing inhibition of 11-KT-mediated repression of Fsh production jointly allows for the concomitant rise of the two main stimulators of spermatogenesis: Fsh and androgens, assuming that Gnrh release is not a limiting factor.

Androgen-mediated up-regulation of gonadotropin gene expression has additional components, one of them involving estrogen signaling. We have found elevated expression of estrogen signaling genes (*esr1*, *cyp19a1b* and *cyp1b1*) in pituitaries of maturing male salmon. Pituitary expression of the estrogen receptor 1 (*esr1*) transcript has been identified in numerous fishes (13) and, in some species, was localized in gonadotropes (34, 59, 106, 107). Similarly, the pituitary of several teleost species, including Atlantic salmon (108), showed brain aromatase gene expression (*cyp19a1b*) or activity. The use of aromatase inhibitors (34, 109) and of aromatizable and non-aromatizable androgens (59, 65) demonstrated the importance of aromatase-mediated conversion of T to E_2_. Since gonadotropes in certain fishes express estrogen receptors and *cyp19a1b*, E_2_ (or T after intracellular conversion to E_2_) could regulate this cell type’s functionality, as hypothesized for medaka Lh-producing cells (59).

Our data on the KEGG pathway enrichment analysis also indicated that, similar to zebrafish (67) and medaka (64), genes related to cell adhesion and extracellular matrix (ECM) are modulated in the pituitary during the start of puberty. This is in accordance with previous studies in mammals, in which components of the ECM were shown to modulate pituitary cell proliferation and hormone secretion (110). As reviewed by Fontaine et al. (4), gonadotrope cells display an extraordinary plasticity at cellular, population and structural level in both fish and mammals, and some physiological processes (including pubertal development) require a remarkable reorganization of the anterior pituitary structure. Matrix remodeling in pituitary cells, through members of the metalloproteinase (MMP) family (i.e. MMP2 and MMP9), was described in human cell lines, an effect that was enhanced after GnRH treatment (111). In this study, we found the modulated expression of the Mmp members *mmp14* and *adam10*, and of *spock1*, a factor known to inhibit MMP2 activity (112). While MMP14 and ADAM10 regulate cell migration and invasion in pituitary adenomas (113, 114), no clear function for SPOCK1 has been described so far in pituitary tissue. However, a genome-wide study has identified *SPOCK1* as a key gene underlying age at menarche in Caucasian woman (112). Therefore, it is tempting to speculate that, also in fish, ECM remodeling may play a role in changes of pituitary cell homeostasis and hormone production accompanying puberty in Atlantic salmon.

Our study also investigated potential effects of different growth factors on pituitary gene expression. The selection of candidates was based on literature available in other fish species in which modulatory effects were reported (8, 115, 116). However, none of the candidates tested provided consistent data, under the experimental conditions investigated, preventing to draw conclusions as to their involvement in regulating gonadotropin gene expression. In particular the lack of effect of activin/inhibin was unexpected. In the European eel (*Anguilla anguilla*) and in goldfish (*Carassius auratus*), activin stimulated pituitary *fshb* mRNA levels (12, 115). In adult mammals, activin of pituitary origin and inhibin of gonadal origin stimulate and inhibit pituitary FSH release, respectively. The stimulatory effect of activin reflects changes in the Gnrh pulse frequency-dependent availability of the activin binding protein follistatin rather than changes in the activin amount (117). While no information is available in fish in this regard, our RNAseq data in salmon show that the expression levels of activin, inhibin and their receptors were low or very low and none of them differentially expressed; the same applies to follistatin (data not shown). Therefore, it appears that activin/inhibin/follistatin signaling is of limited relevance in salmon before and at the beginning of puberty. Considering inhibin, finally, recent genetic experiments in zebrafish suggest that this competitive inhibitor of activin is not of critical relevance for male puberty or adult spermatogenesis (118).

**Figure 7:**
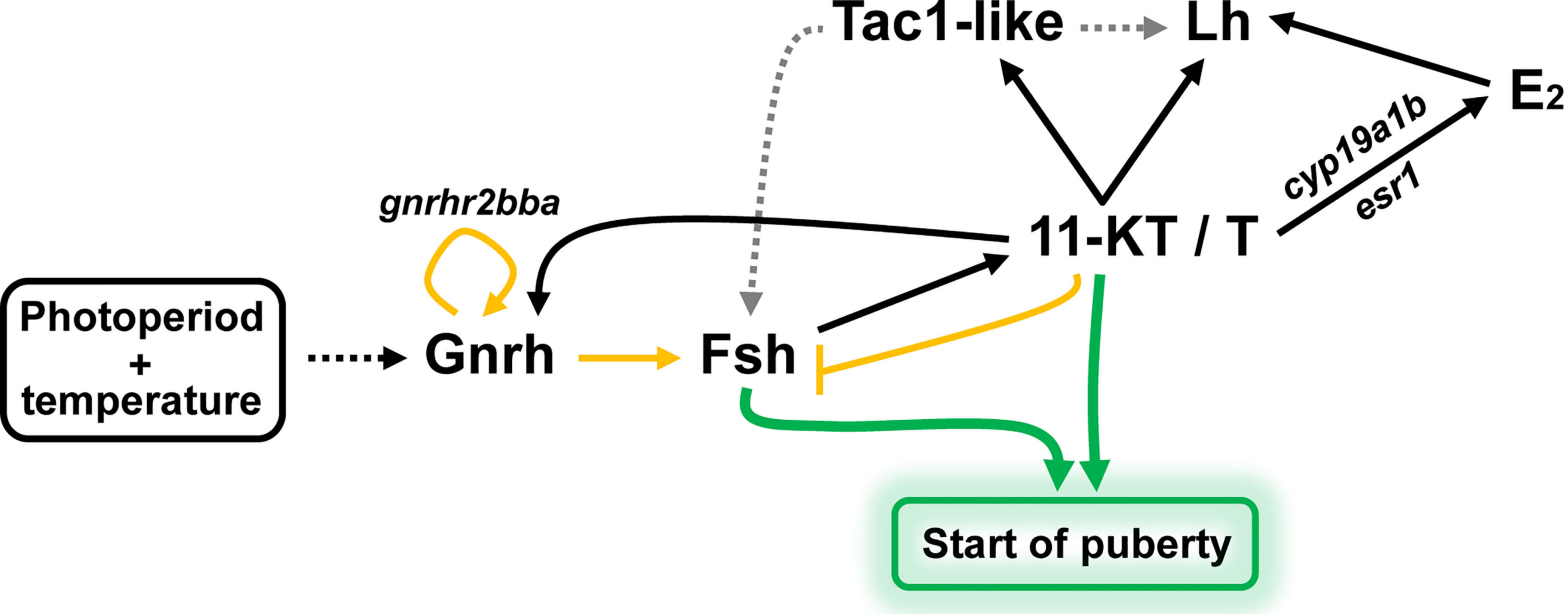
Schematic illustration summarizing the regulation of pituitary Fsh/*fshb* expression and production at the, experimentally induced, onset of puberty in male Atlantic salmon. Described effects are indicated by solid lines, while dashed lines denote no experimental evidence reported here but previously demonstrated in other studies. Yellow lines highlight effects that are potentially limited in time: *in vivo* response to stimulatory conditions blunts both, the stimulatory effect of Gnrh on *fshb* and *gnrhr2bba*, and the inhibitory effect of 11-KT on *fshb* transcript levels. Gnrh, gonadotropin-releasing hormone; Fsh, follicle-stimulating hormone; Lh, luteinizing hormone; 11-KT, 11-ketotestosterone; T, testosterone; E_2_, 17β-estradiol; Tac1-like, protachykinin-like; *gnrhr2bba*, gonadotropin-releasing hormone receptor 2bba; *cyp19a1b*, brain aromatase; *esr1*, estrogen receptor 1.

We have summarized our main findings and integrated them with literature data in **Figure 7**. We assume that exposing immature males to stimulatory environmental conditions can trigger the release of Gnrh. The pituitary responds by releasing the at this time dominating gonadotropin Fsh, resulting in an activation of Leydig cell steroid production, including 11-KT and T. Gnrh also increases pituitary Gnrh receptor expression, but this response is restricted to immature fish that had not been exposed to stimulatory environmental conditions yet, and therefore may represent a short-lived positive feedback loop to initiate the maturational response. Gnrh furthermore increased pituitary *fshb* transcript levels, a response also seen after exposure to stimulatory conditions, but that disappeared with the appearance of type B spermatogonia in the testis, i.e. may also be restricted to the initial phase of puberty. This developmental transition also appeared relevant for the inhibitory effect of 11-KT on *fshb* transcript levels, considering it was only apparent as long as the testes did not contain type B spermatogonia yet. Since 11-KT always (i.e. irrespective of the maturational status and the environmental conditions) increased pituitary Gnrh receptor expression, this setting may result initially in a somewhat limited, but then soon in a more important increase in Fsh and hence androgen production, jointly further supporting the progress of pubertal spermatogenesis. Both, 11-KT and T *via* the androgen receptor and T after conversion to E_2_ *via* the estrogen receptor, increase Lh production that, however, will not be released until approaching the actual spawning season. Finally, both androgen receptor and estrogen receptor ligands always strongly increased pituitary *tac1-like* gene expression in a selective manner, considering that other members of the Tac signaling system were not identified as DEGs by RNAseq. Tac-related neuropeptides potentially stimulate gonadotropin production, and this may apply to peptides derived from the *tac1-like* gene as well. Overall, it appears that in our experimental model the regulatory mechanisms sensitive to photoperiod and temperature mainly use Gnrh signaling to trigger a network of downstream processes. However, genetic evidence suggests that even in the complete absence of Gnrh signaling, zebrafish can still reproduce, demonstrating that the pathways highlighted by our studies are not of the ‘must be taken’ but rather of the ‘can be taken’ category, as has been stressed recently (5, 96) in the light of alternative signaling routes.

## Data Availability Statement

The datasets presented in this study can be found in online repositories. The names of the repository/repositories and accession number(s) can be found in the article/**Supplementary Material**.

## Ethics Statement

The animal study was reviewed and approved by the Norwegian Animal Research Authority (NARA, permit number 5741) and the use of these experimental animals was in accordance with the Norwegian Animal Welfare Act.

## Author Contributions

DC, KS, OY, SO, PV, and JB performed the experiments. DC, KS, EK-S, OY, BN, SO, PV, JB, LK, EA, AW, PF, and RS analyzed and contributed to the interpretation of the results. RE, JB, EA, AW, TH, PF, and RS conceived the project, secured funding, and provided the supervision. DC, AW, PF, and RS wrote the manuscript. All authors contributed to the article and approved the submitted version.

## Funding

This research was financed with resources from The Research Council of Norway (POSTSMOLTMAT project, No. 254870), the EU Seventh Framework Programme *via* the AQUAEXCEL project (No. 262336), and the European Union’s Horizon 2020 research and innovation programme under grant agreement No 652831.

## Conflict of Interest

The authors declare that the research was conducted in the absence of any commercial or financial relationships that could be construed as a potential conflict of interest.

## Publisher’s Note

All claims expressed in this article are solely those of the authors and do not necessarily represent those of their affiliated organizations, or those of the publisher, the editors and the reviewers. Any product that may be evaluated in this article, or claim that may be made by its manufacturer, is not guaranteed or endorsed by the publisher.
