# Supplementary material for: Pituitary Gonadotropin Gene Expression During Induced Onset of Postsmolt Maturation in Male Atlantic Salmon: In Vivo and Tissue Culture Studies
Source: Front Endocrinol (Lausanne). 2022 Mar 15;13:826920. doi: 10.3389/fendo.2022.826920 (PMC8964956; doi:10.3389/fendo.2022.826920)
Supplement: Supplementary file 2 [file Presentation_1.pdf]

Supplementary Material

1 Supplementary Data

Supplementary Data 1. Complete list of differentially expressed genes identified by RNAseq (N = 5; p-adjusted < 0.001).

The table is attached as a separate file.

2 Supplementary Figures and Tables

2.1 Supplementary Figures

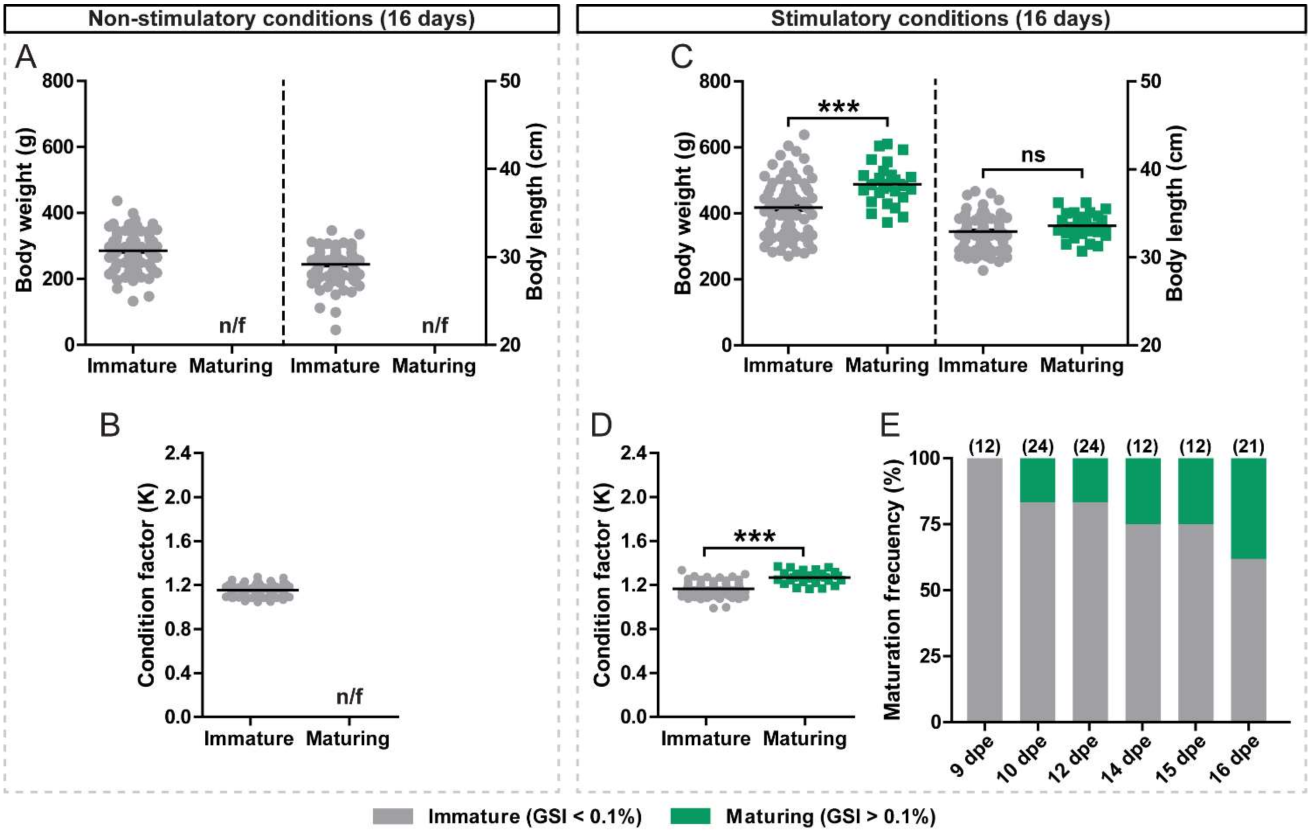

Supplementary Figure 1. Evaluation of medium-term non-stimulatory and stimulatory conditions in male salmon: effects on different biological parameters *in vivo*. (A-E) Body weight and length (A and C), condition factor (B and D) and maturation frequency (E) in immature and maturing male postsmolts after exposure to non-stimulatory (12 hours dark/12 hours light, and 16°C) and stimulatory conditions (constant light and 16°C) for 16 days. In A-D, data are expressed as mean ± SEM (N = 26-69; ***, $p < 0.001$). n/f, not found. ns, no significant differences between groups. In E, dpe indicates days post starting exposure to stimulatory conditions, and numbers in brackets show the number of fish studied at each time point.

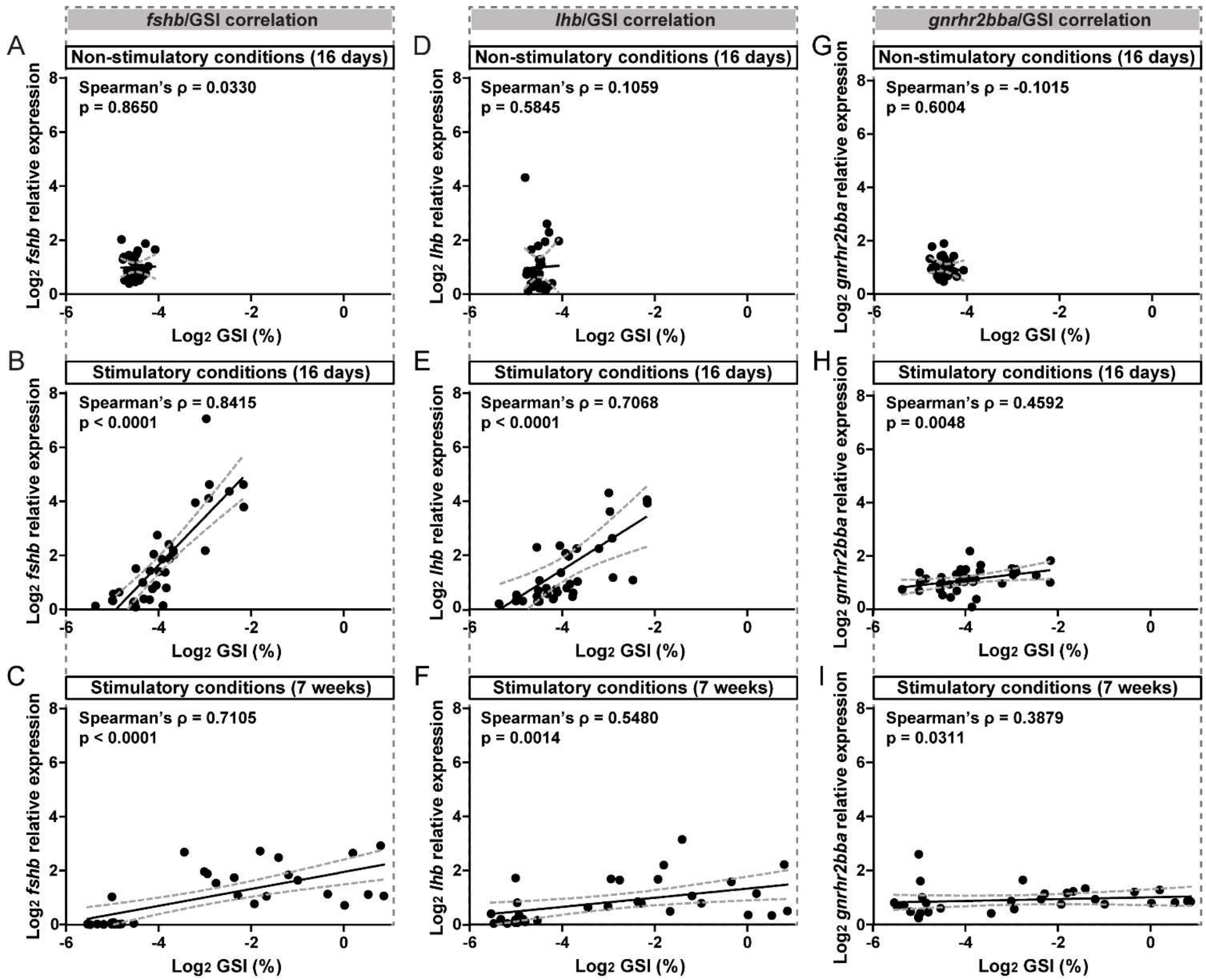

Supplementary Figure 2. Correlations between gonado-somatic index (GSI) and gonadotropin gene expression. (A-I) Comparison of changes in GSI and *fshb*, *lhb* and *gnrhr2bba* expression values derived from different experiments. While no correlation was found in male salmon kept under non-stimulatory conditions (12 hours dark/12 hours light, and 16°C; A, D and G), GSI significantly correlates to the expression levels of selected gonadotropin genes in male postsmolts exposed to both medium- (16 days; B, E and H) and long-term (7 weeks; C, F and I) stimulatory conditions (constant light and 16°C). The number of GSI-gene expression pairs ranges between 29 and 36.

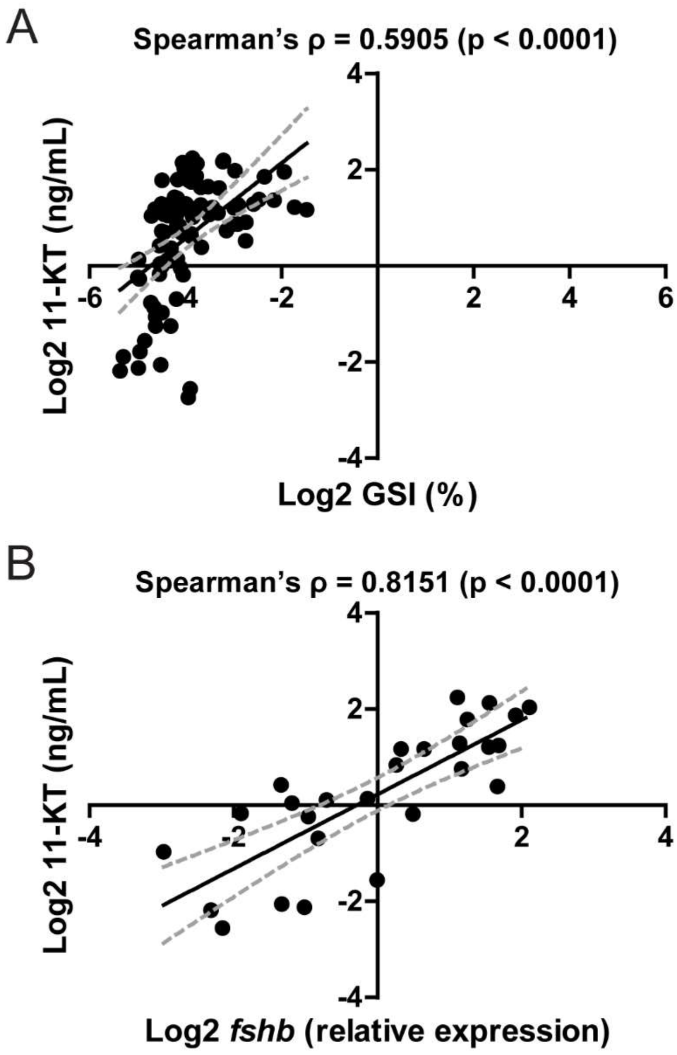

Supplementary Figure 3. Correlation of plasma 11-ketotestosterone (11-KT) concentration to gonado-somatic index (GSI; **A**) and *fshb* gene expression (**B**) in male postsmolts exposed to stimulatory conditions (constant light and 16°C) for 16 days. The number of 11-KT-GSI and 11-KT-*fshb* expression pairs ranges between 89 and 27, respectively.

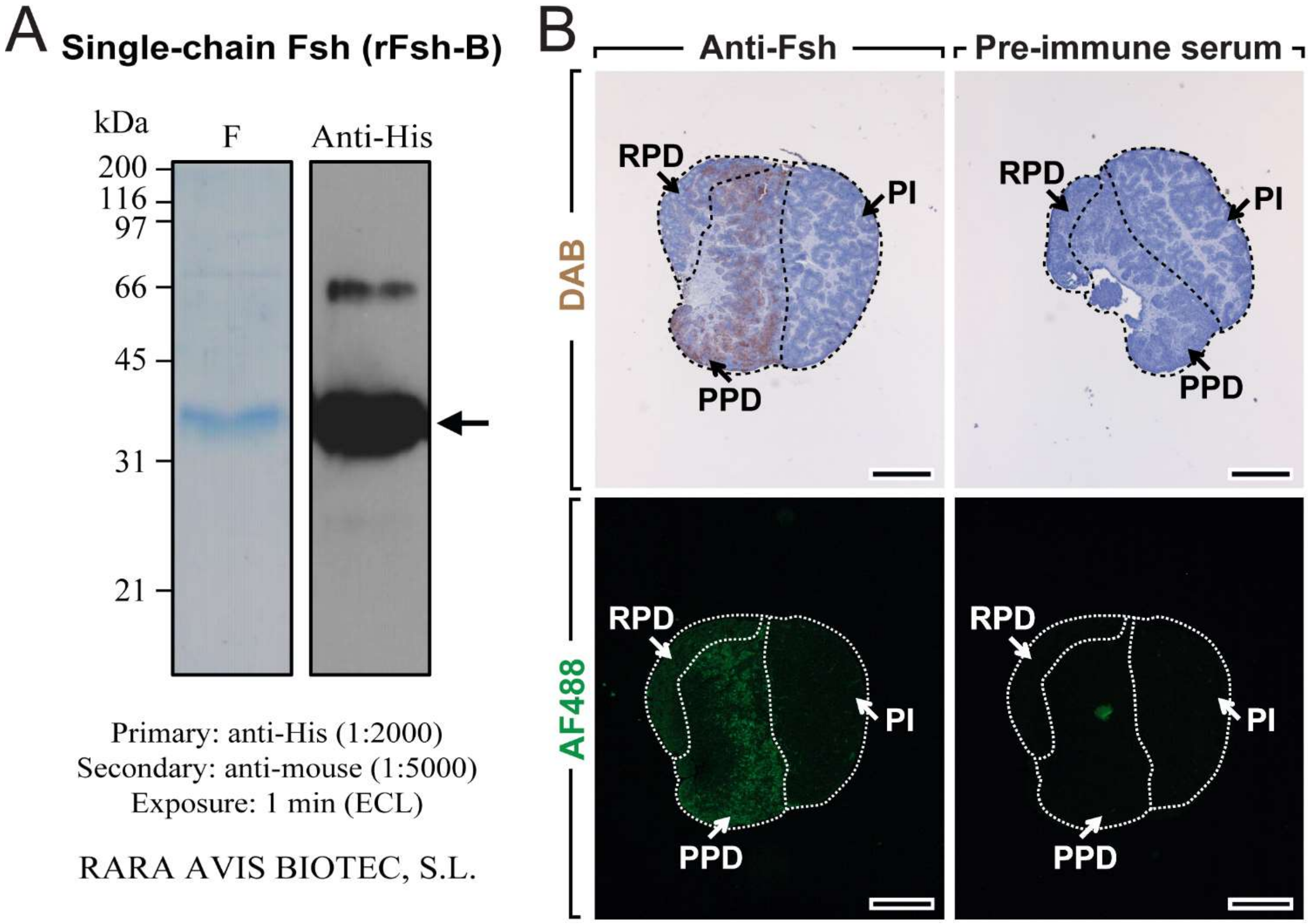

Supplementary Figure 4. Production of single-chain recombinant protein for Atlantic salmon Fsh (rFsh-B) and localization of Fsh in male salmon pituitaries. (A) Representative immunoblot of rFsh-B using an anti-His antibody (right panel), as reported by Rara-Avis (Valencia, Spain). Black arrow indicates the rFsh-B band. The recombinant protein contained a TEV protease cleavage site followed by a 6X His-Tag (used for affinity purification) at the C-terminus of the protein. The left panel shows a collected fraction (F) with Fsh recombinant protein after IMAC purification. Molecular mass markers (kDa) are shown on the left. ECL, enhanced chemiluminescence product for Western blot detection. **(B)** Immunolocalization of DAB- and fluorescent-stained Fsh protein in serial sections (sagittally oriented; anterior to the left) of salmon pituitaries using the Atlantic salmon Fsh antiserum generated in this study (left panels). Negative controls (pre-immune serum) for Fsh immunostaining showed no specific staining (right panels). DAB, peroxidase (HRP) substrate kit; AF488, Alexa Fluor 488; PPD, *proximal pars distalis*; PI, *pars intermedia*; RPD, *rostral pars distalis*. Scale bar = 500 µm.

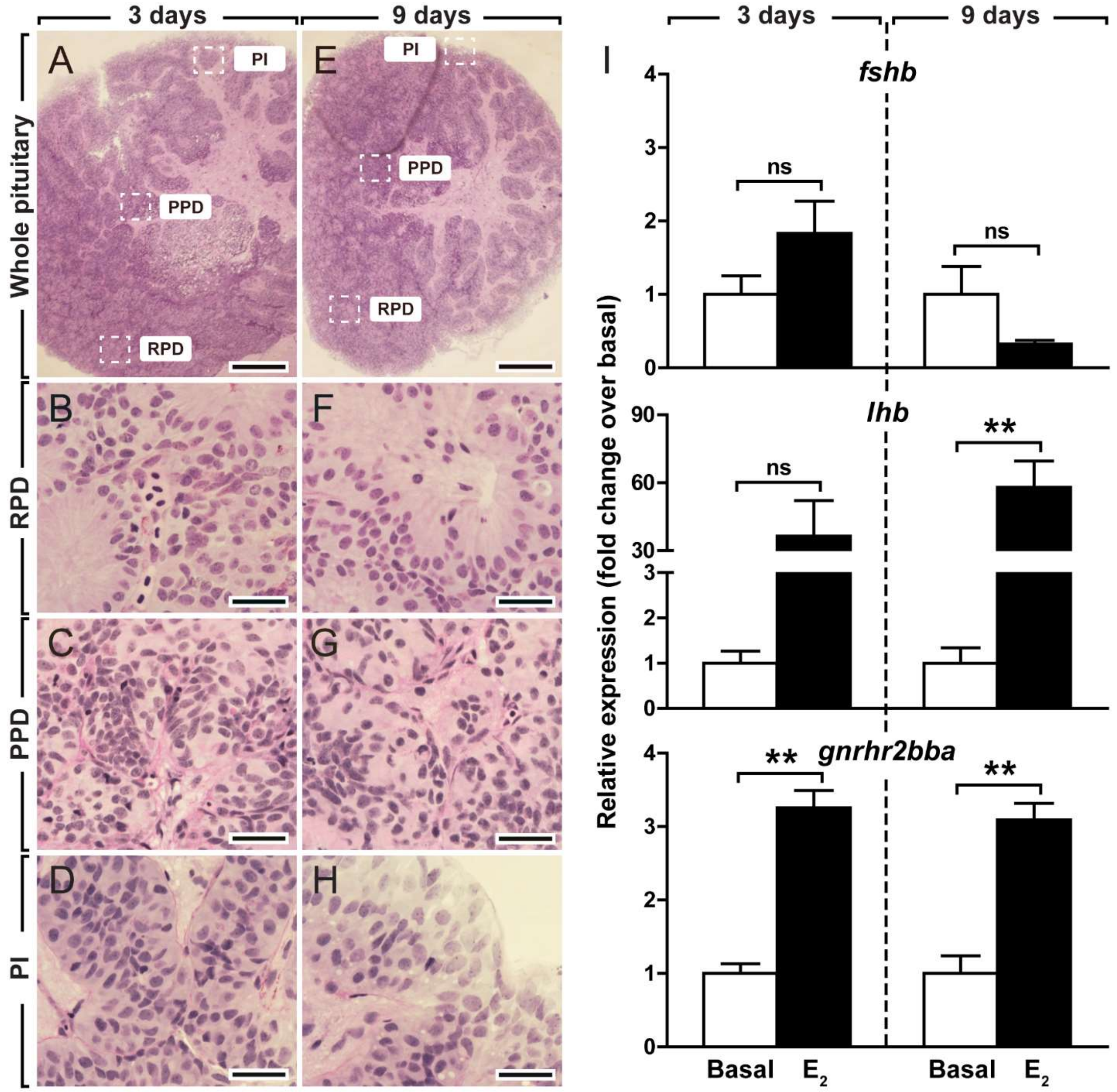

Supplementary Figure 5. Evaluation of morphological and functional integrity of salmon pituitaries after *ex vivo* organ culture. (A-H) Morphological analysis of pituitaries collected from immature male postsmolts exposed to normal photoperiod conditions and 16°C for 16 days, and subsequently cultured *ex vivo*. Sagittal sections (anterior to the left) show the different parts of the pituitary at low magnification (A, E; scale bar = 200 μm), and cell types in the different boxed areas after 3 (B-D) and 9 days of incubation (F-H) at ~15°C at high magnification (scale bar = 30 μm). PPD, *proximal pars distalis*; PI, *pars intermedia*; RPD, *rostral pars distalis*. **(I)** *Ex vivo* estrogen treatment effect on gonadotropin gene expression. Expression levels of *fshb*, *lhb* and *gnrhr2bba* in pituitaries collected from immature postsmolts kept under standard photoperiod conditions and 16°C for 16 days, and subsequently incubated *ex vivo* for 3 and 9 days in the absence or presence of 100 ng/mL 17β-estradiol (E_2). Data are shown as mean fold change ± SEM (N = 3-6; **, $p < 0.01$) and expressed relative to the control basal group, which is set at 1. ns, no significant differences between groups.

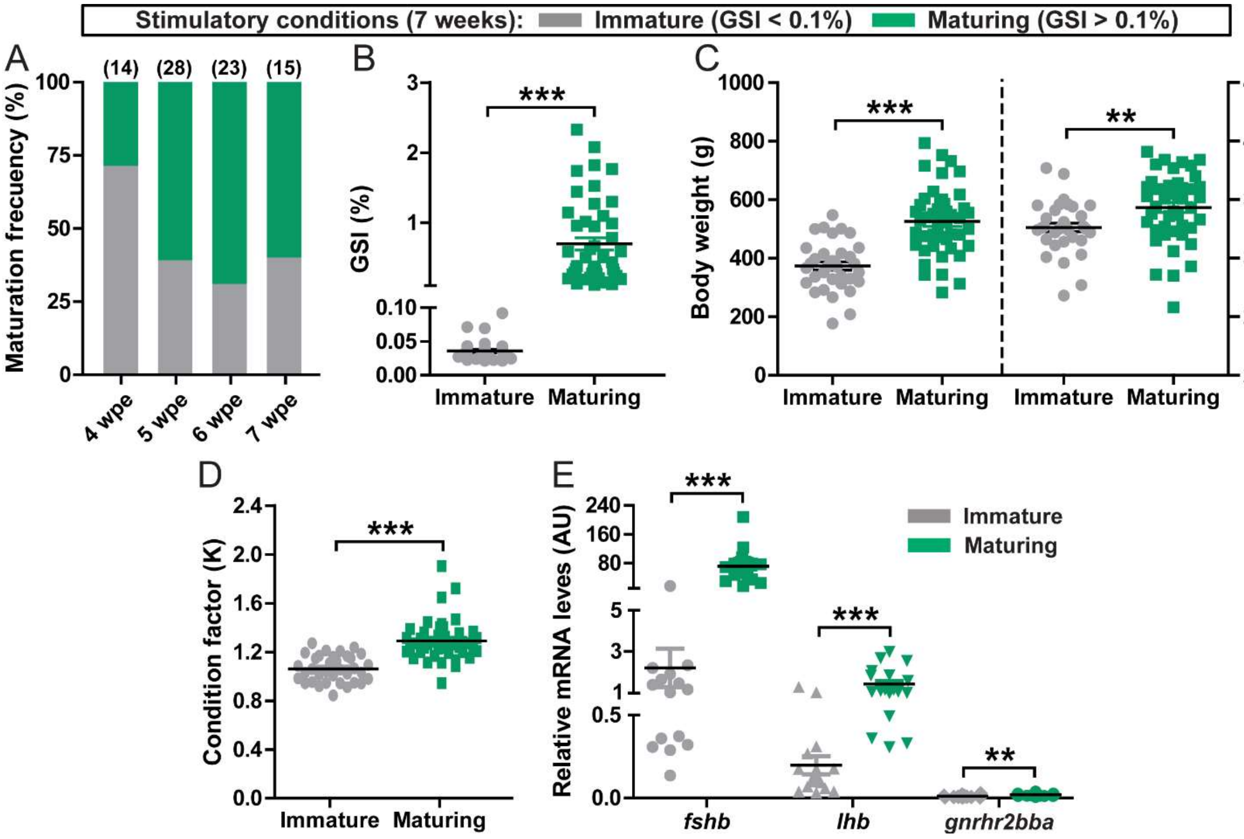

Supplementary Figure 6. Evaluation of long-term stimulatory conditions in male salmon: effects on different biological parameters and pituitary gene expression *in vivo*. (A-D) Maturation frequency (A), gonado-somatic index (GSI; B), body weight and length (C) and condition factor (D) in immature and maturing male postsmolts after exposure to stimulatory conditions (constant light and 16°C) for 7 weeks. In A, wpe indicates weeks post starting exposure to stimulatory conditions, and numbers in brackets show the number of fish studied at each time point. Data are expressed as mean ± SEM (N = 35-46; **, p < 0.01; ***, p < 0.001). **(E)** Expression levels of selected pituitary genes in immature and maturing fish kept under stimulatory conditions for 7 weeks. Results are shown as mean fold change ± SEM (N = 15-23; **, p < 0.01; ***, p < 0.001) and expressed relative to the immature group, which is set at 1.

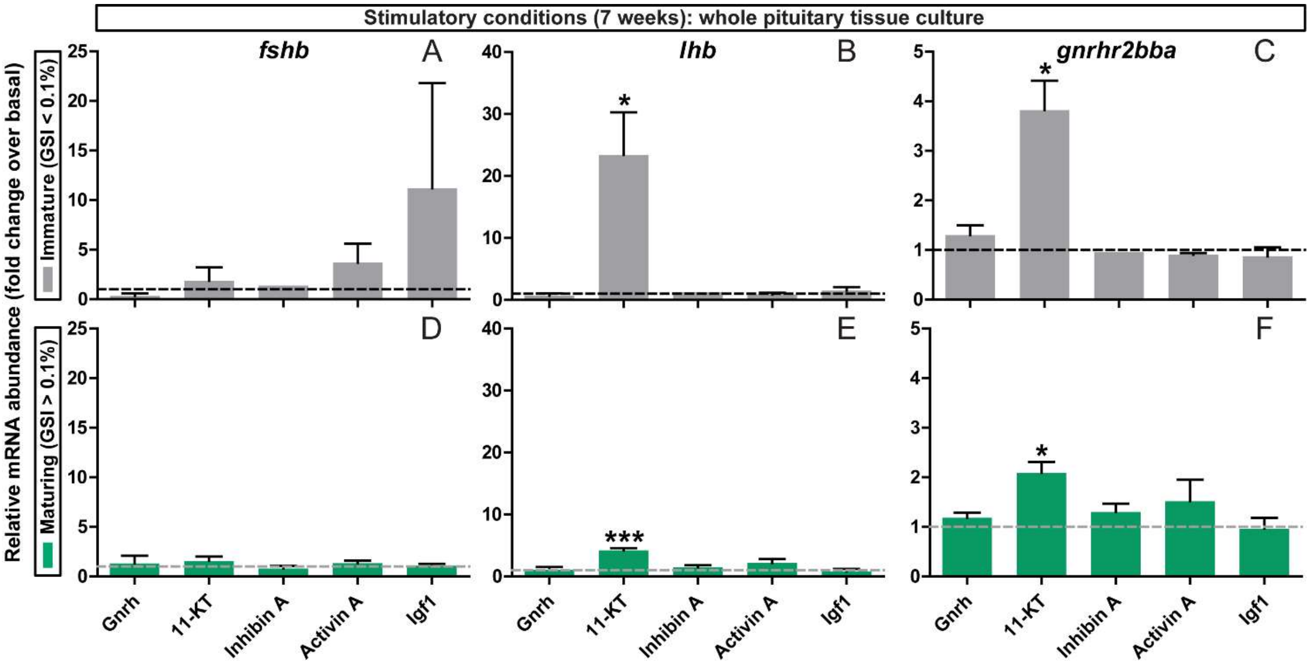

Supplementary Figure 7. ***Ex vivo*** **effects of potential regulators on selected pituitary genes: long-term stimulatory conditions. (A-F)** Expression levels of *fshb*, *lhb* and *gnrhr2bba* in pituitaries collected from immature (A-C; upper row) and maturing (D-F; lower row) postsmolts exposed to stimulatory conditions (constant light and 16°C) for 7 weeks, and subsequently incubated *ex vivo* for 9 days in the presence of various potential regulators of pituitary gene expression. Results are shown as mean fold change ± SEM (N = 3-6; *, $p < 0.05$; ***, $p < 0.001$) and expressed relative to the control basal condition, which is set at 1 (dashed line).

2.2 Supplementary Tables

Supplementary Table 1. Primers used for gene expression studies by qPCR analysis.

Gene annotation	Gene symbol	Primers	Primer sequence (5'→ 3')	PCR efficiency (%)
Brain aromatase (LOC106584746)	*cyp19a1b*	Fw<br>Rv	CTGGGTCCAATCCTGTCCTA<br>TCCAGACCCGAACCATACTC	103.05
Elongation factor 1 alpha (LOC100136525)	*ef1a*	Fw<br>Rv<br>Probe	CCCCTCCAGGACGTTTACAAA<br>CAGACGGCCCACAGGTACA<br>ATCGGTGGTATTGGAAC	(1)
Estrogen receptor 1 (NM_001123592.1)	*esr1*	Fw<br>Rv	TAGGAAGAGCTGCCAAGCAT<br>CGCTTGTCCTTCCTGAGAAC	97.04
Gonadotropin subunit beta-1 (LOC100136362)	*fshb*	Fw<br>Rv<br>Probe	TCACGGAGGCATCACCATCA<br>GCTCTTGGCAACGGGTATGA<br>ACCTGCGCCGGCCTGTGC	(2)
Gonadotropin-releasing hormone II receptor-like (LOC106561195)	*gnrhr2bba*	Fw<br>Rv<br>Probe	TCAACCCACTGGCGATCAAT<br>CGTGATGGTCACACTGTGGAATA<br>AGTGTGATTCTGTCTGTTCCCCAGATGCTG	(3)
Gonadotropin II beta subunit (AF146151)	*lhb*	Fw<br>Rv<br>Probe	GTGCTTAGTCATTCAAACCCCTATCT<br>ACGGTGGAAAATGGGCTCTT<br>CTGCGTCACCAAGGAGCCGGTTT	(2)
Somatolactin beta (LOC100196589)	*smtlb*	Fw<br>Rv	ATGAACCTGGCACAAGGAGT<br>GGTAGTCTGCCAGGCTCTTG	91.92
Protachykinin-like (LOC106582146)	*tac1-like*	Fw<br>Rv	TGGCCACAATCTGAGCAATA<br>TTTTGCAGAGCCTCTTTTCC	99.54
Thyrotropin subunit beta-like (LOC106572976)	*tshbb*	Fw<br>Rv	TGTGTGCGTGTGTATGATGG<br>ACTGCAGATGGTGGTGTTGA	91.18

Fw, forward; Rv, reverse; (1) First validated and published by Olsvik et al. (2005), (2) Andersson et al. (2013) and (3) Melo et al. (2014), respectively.

References

1. Olsvik PA, Lie KK, Jordal AE, Nilsen TO, Hordvik I. Evaluation of potential reference genes in real-time RT-PCR studies of Atlantic salmon. BMC Mol Biol. 2005;6:21.

2. Andersson E, Schulz RW, Male R, Bogerd J, Patina D, Benedet S, et al. Pituitary gonadotropin and ovarian gonadotropin receptor transcript levels: seasonal and photoperiod-induced changes in the reproductive physiology of female Atlantic salmon (Salmo salar). Gen Comp Endocrinol. 2013;191:247-58.

3. Melo MC, Andersson E, Fjelldal PG, Bogerd J, Franca LR, Taranger GL, et al. Salinity and photoperiod modulate pubertal development in Atlantic salmon (Salmo salar). J Endocrinol. 2014;220(3):319-32.
